# Exploring miniature insect brains using micro-CT scanning techniques

**DOI:** 10.1038/srep21768

**Published:** 2016-02-24

**Authors:** Dylan B. Smith, Galina Bernhardt, Nigel E. Raine, Richard L. Abel, Dan Sykes, Farah Ahmed, Inti Pedroso, Richard J. Gill

**Affiliations:** 1 Department of Life Sciences, Imperial College London, Silwood Park, Buckhurst Road, Ascot, Berkshire, SL5 7PY, UK; 2 School of Biological Sciences, Royal Holloway University of London, Egham, Surrey, TW20 0EX, UK; 3 Department of Surgery & Cancer, Imperial College London, MSk Laboratory, London, W6 8RP, UK; 4 Mechatronics, School of Engineering, FHNW, Gründenstrasse 40, 4132 Muttenz, Switzerland; 5 School of Environmental Sciences, University of Guelph, Guelph, Ontario, N1G 2W1, Canada; 6 Core Research Laboratories, Natural History Museum, Cromwell Road, London, SW7 5BD, UK; 7 Bio-Computing & Applied Genetics Division, Centre for Systems Biotechnology, Fraunhofer Chile Research Foundation, Avenida M. Sánchez Fontecilla 310, 7550296, Chile

## Abstract

The capacity to explore soft tissue structures in detail is important in understanding animal physiology and how this determines features such as movement, behaviour and the impact of trauma on regular function. Here we use advances in micro-computed tomography (micro-CT) technology to explore the brain of an important insect pollinator and model organism, the bumblebee (*Bombus terrestris*). Here we present a method for accurate imaging and exploration of insect brains that keeps brain tissue free from trauma and in its natural stereo-geometry, and showcase our 3D reconstructions and analyses of 19 individual brains at high resolution. Development of this protocol allows relatively rapid and cost effective brain reconstructions, making it an accessible methodology to the wider scientific community. The protocol describes the necessary steps for sample preparation, tissue staining, micro-CT scanning and 3D reconstruction, followed by a method for image analysis using the freeware SPIERS. These image analysis methods describe how to virtually extract key composite structures from the insect brain, and we demonstrate the application and precision of this method by calculating structural volumes and investigating the allometric relationships between bumblebee brain structures.

In spite of the pivotal role of the brain in controlling animal behaviour, very little is still understood about how its structural and functional complexity determines the diversity of behaviours observed^1^. Investigations of brain structure, and its role in determining specific behavioural traits, have propelled critical developments in imaging methodology, such as magnetic resonance imaging (MRI) and X-ray computed tomography (CT). For example, CT scanning has highlighted that variation in volume, shape and density of particular human brain regions can be correlated with phenotypic syndromes and diseases^2^,^3^. The implementation of such technologies to the study of smaller organisms with tiny brains (such as insects), however, is notably lacking^4^,^5^,^6^. A major barrier to progress in this area is that imaging at this small scale is a more challenging task due to problems with low image resolution and the practicalities of manipulating, preparing and observing miniature composite structures^4^,^7^.

So why study insect brains? Despite their comparatively small size, insect brains are capable of rapidly detecting and responding to a plethora of diverse stimuli in a wide range of sensory modalities, facilitating their global ecological success and establishing them as an essential model system for cognitive biology and neuroscience^8^,^9^,^10^,^11^,^12^,^13^. Although insect brains are smaller and simpler than their vertebrate counterparts, there is increasing evidence that insect cognitive performance can be impressive^11^,^14^,^15^,^16^,^17^,^18^,^19^,^20^,^21^,^22^,^23^,^24^,^25^,^26^,^27^. For instance, foraging insects must learn and memorise navigation routes in complex landscapes requiring the ability to detect, distinguish and integrate a multitude of chemical, visual, landmark and celestial cues^11^,^13^,^25^,^27^,^28^,^29^,^30^,^31^,^32^. Therefore, knowledge of insect brain structure allows us to understand how comparatively small (and simple) brains can generate complex patterns of behaviour and act as a gateway to understanding more complex brains and their evolutionary development^8^,^13^,^16^,^33^,^34^. Indeed, variation in the volume of brain regions (examined using histological techniques) has been reported to be linked to differences in innate responses to stimuli^35^, age/experience related behavioural transitions^36^,^37^,^38^, behavioural syndromes^39^,^40^ and rates of learning and performance in cognitive tasks^41^,^42^. Yet, there remains much to discover about how insect brain structure relates to individual behaviour^1^,^16^,^39^. Closing such a fundamental knowledge gap requires the development of new imaging protocols and the application of novel strategies to measure, record and robustly quantify aspects of brain morphology across multiple individuals.

The majority of investigations into insect brain morphology have used traditional histological techniques (see Supplementary Tables 1 and 2 for a list of representative studies). Producing 2D images with these techniques requires invasive dissection for image preparation followed by relatively time-consuming fixing and physical tissue slicing using a microtome^43^. Typically brain samples prepared using this method can suffer tissue distortion, desiccation and permanent damage, leading to biased measurements that impede accurate quantification of morphology^44^,^45^,^46^,^47^. Magnetic resonance and confocal microscopy imaging have been used to study insect brains, eliminating the need for tissue slicing and/or staining, but these approaches suffer from comparatively low resolution and semi-destructive treatment of the samples^48^,^49^,^50^,^51^. The development and application of micro-CT to investigate the small brains of insects is a significant milestone towards collecting unprecedented data and insight into brain structure^52^,^53^,^54^,^55^ and offers the potential for studies linking variation in brain structure with behavioural differences. The landmark study of Ribi *et al.* (2008)^52^ showcased the use of micro-CT in exploring insect tissues, specifically in the honeybee (*Apis mellifera*) brain. After removing the head musculature and salivary glands, the authors used an osmium-based staining method to enhance contrast and showed that the main brain structures could be distinguished from other tissues. Other authors have shown that the integration of brain reconstructions can provide a powerful method for producing structural atlases of standardised insect brain maps that facilitate fast, semi-automatic analysis (region annotation, segmentation and volume extraction) across multiple individuals^4^,^7^,^49^,^51^,^55^,^56^,^57^,^58^. While Ribi *et al.* (2008)^52^ provided a proof-of-concept that high resolution imaging of insect brains is possible, brain atlases to date have typically been constructed using lower resolution and invasive techniques. Therefore, developing protocols to apply high-resolution imaging (such as micro-CT) coupled with segmentation, for quantitative volumetric and 3D morphological analyses, would improve our ability to understand the intricate details of brain morphology, and link this structural variation with organismal function through comparative analyses, both within and among species, across a range of ontologies and life histories.

Here we describe a methodology that builds on the pioneering work of Ribi *et al.*^52^, to allow exploration of the composite structure of small-scale soft tissues without disturbing the insect brain within the head case, hence retaining its natural stereo-geometry and minimising any potential tissue destruction. By exploring the brain of a common European bumblebee (*Bombus terrestris*) we outline a relatively easy-to-use protocol, that reduces the effort needed to prepare samples for high resolution micro-CT scanning, and couple this with the application of open source (freeware) visual analysis software SPIERS to generate non-destructive, 3D reconstructed brain images at relatively high resolution (achieving a standardised 4.6 μm voxel size per scan – an improvement on 7 μm reported by previous studies^52^). Furthermore, we demonstrate an application of this method by segmenting and virtually extracting five of the primary brain composite structures for 19 individual bees. We present quantitative volumetric measurements for each of the five brain structures: mushroom bodies (MBs), antennal lobes (ALs), medullas (Mes), lobulas (Los) and the central body (CB) (see Supplementary Table 3 for brief description of each structure). This study provides an appreciable number of samples, allowing us great insights into the degree of variation in brain morphology and how to achieve the appropriate level of statistical power required for future comparative analyses. As a result this method provides a critical step towards detailed geometric morphometric studies and investigations of links between brain-structural variation and organismal function in insects.

## Results and Discussion

Working towards the objectives of attaining high resolution images and precise 3D morphological measurements of insect brain morphology, we addressed several of the key challenges faced by researchers when attempting to use X-rays to image small brain structures: a) sample preparation: we optimised a protocol for specimen preparation, using a non-hazardous dye and simple staining procedure, that produces images with appropriate contrast for distinguishing soft tissues of interest for downstream image analyses; b) scanning of specimens: our protocol used settings to scan multiple individuals at once to reduce cost and machine running time while still producing high quality images; c) image segmentation and validating the biological veracity of the data: we have developed a useful, freeware-based, protocol for image analysis to segment brain regions with high confidence, capable of systematically providing precise and repeatable measures of morphological features.

Generating accurate visualisations of independent brain structures critically requires the ability to differentiate multiple tissues based on density, shown by the degree of staining that provides effective contrast enhancement. To facilitate stain perfusion throughout the brain tissues, we removed the front cuticle of the bee head-case without disturbing the brain^52^ (see Supplementary Fig. 1a). Standardizing the staining process involved studying the dynamics of tissue staining over time (i.e. the rate at which specimens take-up the stain, and contrast provided when repeatedly imaged). We tested the efficacy of three staining solutions previously used on insect tissues: iodine (I), uranyl acetate (UA) and phosphotungstic acid (PTA: see Online Methods for details). We excluded osmium-tetroxide, used by Ribi *et al.* (2008), from the comparison because its toxicity profile precludes widespread general use. To examine the effectiveness of staining, each head was micro-CT scanned once per day for specified days over a nine-day period (see Methods). We found that seven days of PTA staining provided the best contrast enhancement and resolution of brain structures, and was therefore chosen as the staining method for bee brains prior to scanning (Supplementary Figs 2–4).

**Figure 1:**
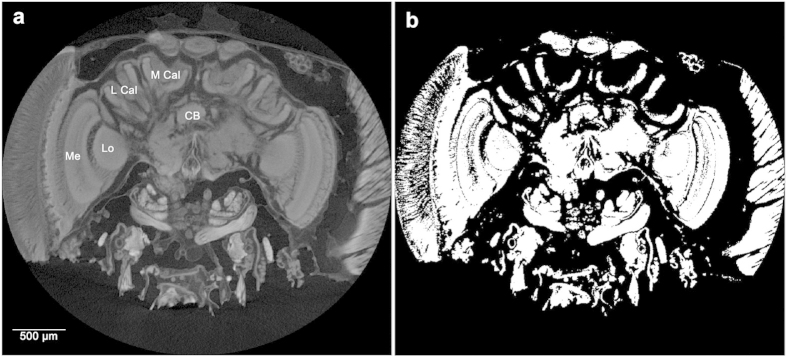
Bumblebee brain micro-CT scan slice. Raw image (**a**) showing optimum orientation and slice plane for viewing brain structures for segmentation, visible structures of interest: lateral calyx (L Cal), medial calyx (M Cal), central body (CB), medulla (Me), lobula (Lo), and (**b**) the optimum threshold for segmentation of the mushroom body calyces of the same slice.

**Figure 2:**
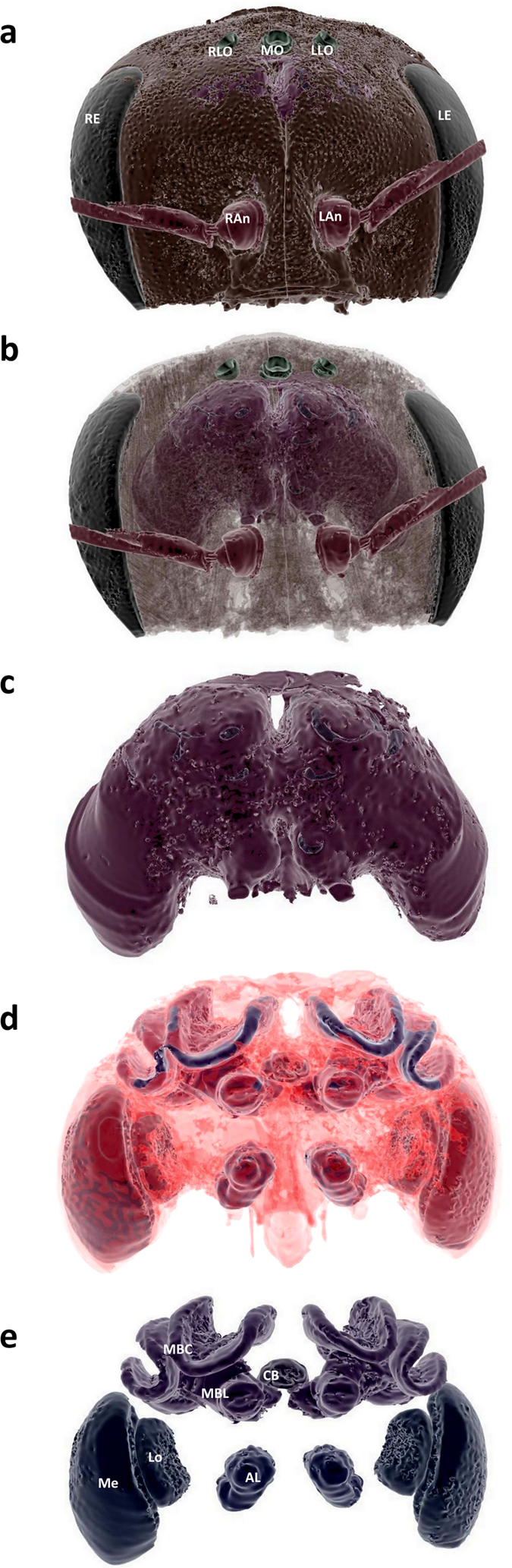
3D rendered views from micro-CT imaging of bee head case, brain and brain structures. (**a**) External head case with independently segmented structures: right eye (RE), left eye (LE) right lateral ocellus (RLO), median ocellus (MO), left lateral ocellus (LLO), right antenna (RAn), left antenna (LAn). (**b**) transparent head case showing brain in situ; (**c**) brain tissue, virtually segmented from head case; (**d**) transparent brain tissue showing brain structures in situ; (**e**) individually segmented brain structures independent of additional brain tissue showing: central body (CB), and one of the pair of lobulas (Lo), medullas (Me), antennal lobes (AL), mushroom body calyces (MBC) and mushroom body lobes (MBL). The images show false colour application to the head and brain structures, and images (**c**–**e**) have been magnified 1.5× in size compared to (**a**,**b**).

**Figure 3:**
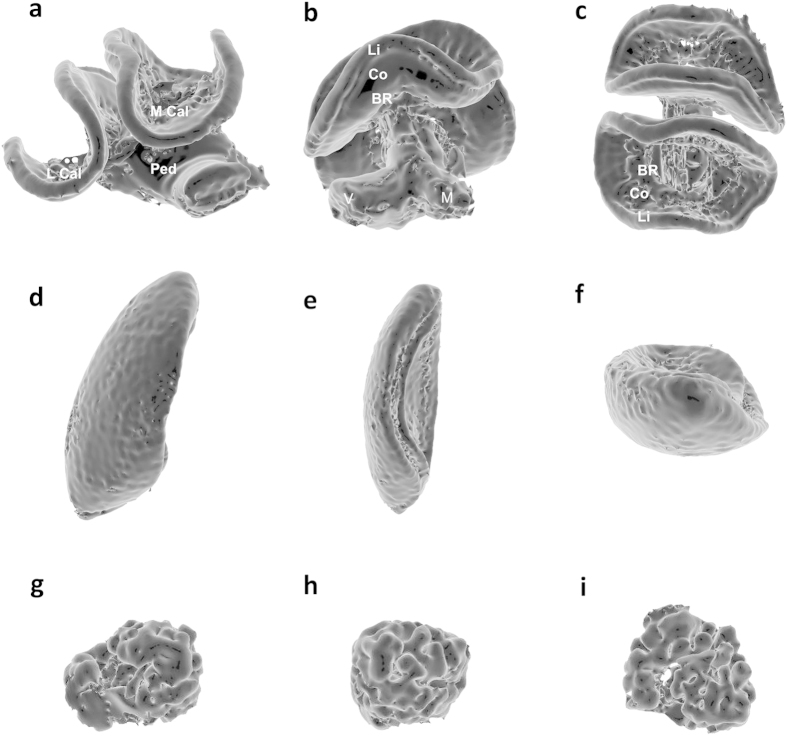
3D SPIERS View renderings from micro-CT imaging of a mushroom body (**a**–**c**), medulla (**d**–**f**) and antennal lobe (**g**–**i**). (**a**) Right mushroom body frontal view highlighting the: lateral calyx (L Cal), medial calyx (M Cal), and pedunculus (Ped); (**b**) medial side view highlighting the: lip (Li), collar (Co), basal ring (BR), vertical lobe (V) and medial lobe (M); (**c**) dorsal view; (**d**) right medulla frontal view; (**e**) medial side view; (**f**) dorsal view; (**g**) right antennal lobe frontal view; (**h**) medial side view; (**i**) dorsal view.

Micro-CT scans were performed using a Nikon Metrology HMX ST 225 system^59^ with a molybdenum target (Supplementary Fig. 5). The raw data for each brain scan were reconstructed using CTPro 2.1 software (Nikon Metrology, Tring, UK) and visualised using VG Studio Max 2.1 (Volume Graphics GmbH, Heidelberg, Germany) for alignment and re-slicing of all the samples along the same optimum orientation plane (see Fig. 1a). For each sample we exported scan images at a standardised voxel size representing the upper limit of the range, which was 4.6 μm (scan resolution ranged between 3.1–4.6 μm). Segmentation and volume analysis of brain structures were carried out using the freeware SPIERS 2.20 (Serial Paleontological Image Editing and Rendering System). Using SPIERS required us to develop a novel protocol to specifically segment insect brains. Individual scans were opened in the form of an 8-bit greyscale bitmap (BMP) image stack (see Supplementary Video 1), and could be viewed in greyscale or binary form (a black and white visualization of the image; Fig. 1b). For segmentation, we first compared two different methods of thresholding on two brain structures (Mes and MB calyces): i) a manual ‘tracing method’ judging by eye; and ii) a second method based on ‘pixel intensity histograms’ (see Methods and Supplementary Results). For both methods we started by converting scan slices to binary threshold images representing the optimum ratio of active white pixels, comprising the structure of interest, and inactive black pixels, for the surrounding tissues (see Online Methods). Structures were then defined by placing looped splines around the active pixels at five slice intervals. Splines were finally interpolated across all slices, between five slice intervals, to form the framework to create a mask around the structure, defining it as an independent object for 3D reconstruction and volumetric analysis (see Figs 2 and 3; Supplementary Video 2). Volumes calculated from the tracing- and pixel intensity histogram methods gave almost identical results for the simpler structure of the medullas. Seventeen of the 19 samples had the same calculated volumes, with a mean difference between the two methods for the 19 brains of just 0.45% (median (IQR) = 0% (0 - 0)). For the more complex mushroom body calyces 10 of the 19 structures had the same volumes, with a mean difference in calculated volumes between the two methods for the 19 brains being 3.8% (median (IQR) = 0% (0 - 5.5); see Supplementary Fig. 6 and Supplementary Table 4). Each method had its pros and cons, with the tracing method potentially suffering from subjective decisions for node placement but benefitting from making common-sense decisions as to what should constitute the tissue of interest, whereas the pixel intensity histogram provides an objective method but may include neighbouring pixels that are not part of the tissue of interest. For this paper, we used the tracing method to calculate volumes of the remaining brain structures.

**Figure 4:**
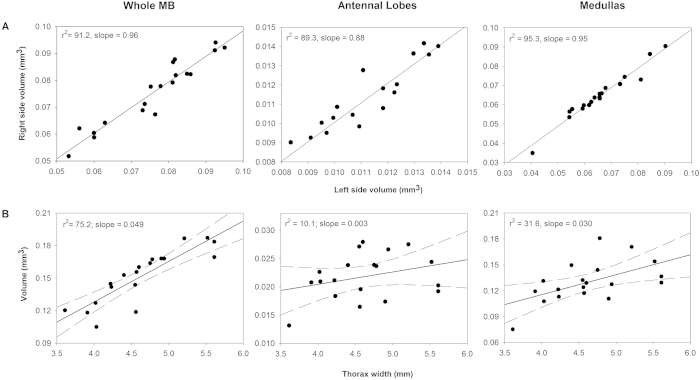
(**A**) Isometric relationship between the calculated volumes (mm^3^) of three paired structures found on the left (x-axis) and right (y-axis) sides of the brain (n = 19), and (**B**) allometric relationship between body size (thorax width (mm)) and total volumes of each of the paired structures combined (n = 19). Using the manual tracing method; (**A**) Fitted linear regression lines are plotted with r^2^ values and slope gradients shown. The very high degree of congruence between the volume of left and right paired structures strongly supports that our method is effective at differentiating and extracting the structural tissue. (**B**) Fitted linear regression lines are plotted with 95% confidence limits (dashed line), and r^2^ values and slope gradients are shown. For the whole mushroom body (MB) we explored the relationship when the lobes and calyces were combined.

We further validated our method of brain segmentation by testing the precision of our calculations. We looked at the similarity of our estimates by repeating full segmentation and volumetric analysis four times for both the left and right medullas from one brain. We found low test re-test repeatability scores of 0.0016 for the left medulla (mean = 0.0745 mm^3^; s.d. = 0.0008, c.v. = 1.08%) and a score of 0.0011 for the right medullar (mean = 0.0751 mm^3^; s.d. = 0.0006, c.v. = 0.74%; see Supplementary Table 5), showing high precision of our volumetric estimates. Secondly, we examined the self-consistency of our volumetric estimates by comparing left and right-paired structures for each brain across all individuals (single structure of the CB was thus excluded). Finding very similar (or the same) paired volumetric calculations would support the precision of our method when assuming that such paired structures typically develop symmetrically. We found strong linear relationships, and low mean percentage differences, between the left and right paired structures (whole MBs: r^2^ = 91.2, %diff .= 3.85%; MB lobes only: r^2^ = 74.8, %diff. = 7.48%; MB calyces only: r^2^ = 93.4, %diff. = 4.10%; ALs: r^2^ = 89.3, %diff .= 4.92%; Mes: r^2^ = 95.7, %diff. = 3.06%; Los: r^2^ = 72.1, %diff .= 6.58%; Fig. 4A, Supplementary Fig. 7 and Supplementary Table 6). The differences between the paired volumes are small and our findings support that our methods can objectively assess the volume of morphologically complex brain structures with relatively high precision. Indeed, the differences we do find likely represent an upper limit of error considering that true asymmetry between brain hemispheres may naturally develop, although the extent of this has not been quantified.

Our results conformed to the general allometric relationships between brain structures and body size found in previous histological studies - a significant positive correlation between predictors of body size and each brain structure (MBs, ALs, Mes and Los), with the exception of the CB^38^,^60^ (Fig. 4B, Supplementary Figs 9 and 10). This general conformity supports the view that our results do not suffer from significant artefacts or misrepresentations of brain tissue. However, our methods should generate structural volumes that more accurately reflect real volumes, whereas histological techniques are likely to suffer from multiple sources of error. For instance, histological techniques estimate structural volumes by using only a subset of microtome slices with each slice being typically thicker than ours (e.g. 10–20 vs 4.6 μm), whereas our methodology incorporates structural tissue across all slices (i.e. mean = 377 slices) into an automated volume calculation. Furthermore, from our optimum thresholding procedure, we can differentiate tissues based on density and thus facilitate the exclusion of extraneous internal tissue from each slice enhancing our ability to examine real representations of these complicated structures, with surfaces textured with grooves, pits, crevices and hollows (Fig. 3). Histological techniques, however, are unable to do this as volumes are calculated by visually tracing the structure of interest for each of the subset of slices, taking this calculated area and then interpolating between the subset of slices. A potential shortcoming of this approach is that it assumes the structure has a uniformly even surface between slices, yet this is unlikely to be true (as shown by our images; Fig. 3). We therefore make the prediction that our volume calculations will be consistently lower than those calculated in previous histological studies of bee brains (see Supplementary Table 1–2). If so, we would further expect that the two case-studies^60^,^61^ we compare to our dataset would overestimate the volume of structures with more complicated shape (in this case the MBs, Mes, ALs and Los are more morphologically complex than CB). Indeed, our results confirmed both of these predictions with all of the structures having lower volumes, with our calculated volumes of the MBs, ALs, Mes and Los being, respectively, 49/69%, 44/53%, 60/56% and 63/59% of the volumes estimated in case-studies^60^,^61^, and the CB being 88/80% of the size (see Supplementary Table 7 for details). Although, further explanations for our smaller estimated structural volumes could be that the previous case-studies examined brain volumes in a different bumblebee species, and/or that our sampled brains were not a mature adult size given we sampled very young bees (only four days old) with little experience and that brain structure volumes have been reported to increase with age by as much as 37%^61^.

Developing and combining the advances in imaging technology^52^,^53^,^54^ with a detailed imaging analysis protocol has allowed us to produce high quality 3D reconstructions of soft tissue samples with high resolution and precision that can be revisited for repeated new exploration, virtual dissection and comparative analyses of tissue structure without the need for additional sample preparation (see Figs 2 and 3; Supplementary Video 2). The methods presented here provide a relatively cost-effective and time-efficient toolkit to enable a wide-range of researchers to explore intra- and inter-individual variability of soft tissues (in this case brain structure), and their links to organismal phenotypes (minimizing sample preparation artefacts), which can be applied to both insects and other invertebrate taxa. Given the important ecosystem services provided by beneficial arthropods (e.g. pollination and bio-control of pests), and recent reports of global pollinator declines, our methodology can help elucidate the factors affecting physiology and behaviour helping us to appropriately assess and mitigate such problems^62^. Indeed, the development of this scanning and image processing protocol is particularly timely as it has great potential for investigating whether exposure to specific stressors, such as disease or agrochemicals, can significantly affect brain development, function and associated key behaviours such as learning, foraging and navigation^63^,^64^,^65^,^66^.

## Methods

### Bumblebee husbandry, sampling and sample preparation

Five bumblebee colonies (*Bombus terrestris*) were obtained from a commercial company (Koppert Biological Systems, the Netherlands). Upon arrival, colonies (each containing a queen and a mean of 95.4 workers (range = 71–127)) were transferred to wooden nest boxes within 24 hours. All colonies were then provided with *ad libitum* pollen and sucrose solution (40/60% sucrose/water). To understand the standing variation in brain morphology and to consider the precision of our measurements, we sacrificed individuals for brain scanning that were all of the same age – four days old. These early-adults had eclosed from their pupal case after the colonies had spent at least 21 days (approx. time taken for a worker to be reared from egg to adult^67^) under the same laboratory setting. Age matched individuals were sacrificed because adult maturation, changes in social environment and increased experience can all have effects on brain morphology^36^,^61^. We monitored colonies twice per day and marked any newly eclosed individuals using numbered Opalith tags (Christian Graze KG, Germany), which consequently allowed us to track the age of each individual throughout the sampling period.

Young bees were used for brain scanning to limit any potential changes in brain structure/size during adult development associated with allocation to different tasks in the colony. Bees were sacrificed by removing an individual from the colony using forceps, and then swiftly decapitating the live individual using a disposable surgery scalpel (Supplementary Fig. 1a). Once cut, the head was fully submerged in a 70/30% ethanol/de-ionised water solution in a 1.5 ml centrifuge tube and stored at 5 **°**C until the staining process was undertaken.

The head case was prepared for staining by removing the front part (just below the antennae; see Supplementary Fig. 1a) with a scalpel under 10× magnification (using a stereomicroscope) ensuring that we did not cut into the brain. The head was then fully submerged in the staining solution in one well as part of a multi-well cell plate. We measured thorax width for each decapitated individual, just above the tegula (see Supplementary Fig. 1b), using a set of digital callipers (accuracy = 10 μm) as this has been shown to be strong estimator of body mass in bumblebees^68^. Thorax width measurements were taken twice per individual and the average taken. We sacrificed 34 bees for staining (n = 6 from one colony for the staining optimization test; n = 28 from five colonies for brain 3D reconstruction).

### Selecting staining conditions

Measures of contrast enhancement to view staining success have been used for imaging animal soft tissue in previous studies^69^ although each have employed differing stains, techniques and preparation. In order to develop an accurate and repeatable protocol for visualising bumblebee brain structures, we first focused on establishing a staining procedure. There are a number of widely used tissue stains including uranyl acetate (UA)^70^,^71^, iodine (I)^69^,^70^,^72^, phosphotungstic acid (PTA)^6^,^69^,^73^ and osmium tetroxide^52^,^71^. For our investigation we examined the effectiveness of: i) 1% I solution (1 mg/ml concentration in 70/30% ethanol/water solution); ii) saturated (0.9–1%) aqueous solution of UA; iii) 0.5% PTA solution (0.5 mg/ml concentration in 70/30% ethanol/water solution). Osmium tetroxide was excluded due to its high toxicity and cost^69^,^74^.

For each optimization test we prepared two heads per stain. Each of the six heads were individually scanned on days 1, 3, 6, 7, 8 and 9 to observe how well the stain penetrated the brain (see Supplementary Fig. 2). *Uranyl acetate* – the brain scans indicated little staining across all nine days. As differentiation of brain regions could not be seen or was very difficult to detect we concluded this to be a poor staining method for soft brain tissue CT scanning. *Iodine* – this permeated the brain more extensively than UA, and was quicker to stain tissue than PTA as it only required around 1–3 days. However, the contrast threshold for iodine was not as good, tissues were not as readily identifiable and edges were less defined compared to PTA stained samples. Moreover, after just six days iodine began to bleed causing very blurry edges and then poor tissue differentiation. PTA showed the highest level of contrast enhancement after seven days of staining of all stains, and in comparison to Iodine we found no evidence of stain bleeding, even after nine days. Additionally, when comparing identical phases of perfusion for each stain, PTA produced superior definition of brain regions compared to Iodine and UA. Therefore, we decided to use seven-day exposure to PTA stain based on the achievable image quality (see Supplementary Fig. 3 and 4). Scans for all staining assays were performed using the following settings: 80–90 kV at 100–110 μA, gain 6 dB, with no noise reduction and no beam hardening corrections, and was balanced by increased projections to 6284 and exposure time of 354 ms to provide a suitable noise to signal ratio. The relative performance of the stains we assessed could vary under different scanner settings, therefore, additional testing of these settings could be further optimized for each stain. For example small decreases to the voltage to align with the excitation edges for tungsten, and to increase the current to increase flux.

### Scanning of specimens

Micro-CT scans were carried out at the *Imaging and Analysis Centre*, London Natural History Museum, using a Nikon Metrology HMX ST 225 system (Nikon Metrology, Tring, UK), with cone beam projection system, four megapixel detector panel, maximum voltage output of 225 kV for the reflection target and a maximum current output of 2000 μA (Supplementary Fig. 5). The focal spot size is 5 μm and the exposure ranges from 0.25–5.6 frames per second. Reconstructed data were visualised in VG Studio Max 2.1 in which samples were rotated and re-sliced along the orientation plane that gave the optimum view for segmentation (Fig. 1a; mean slices per brain = 377, range = 294–580; see Supplementary Video 1). We micro-CT scanned the seven-day PTA stained brains by scanning two heads per scan run by inserting two heads inside a plastic straw, held firmly in place by rigid foam at each end and separated by tissue paper, before being placed in the scanner. Due to the number of scans required we needed to scan over a seven day period, therefore to ensure all bee brains were scanned at a standardised number of days after the first day of staining, we respectively staggered the staining start date.

From the 28 brains that were prepared and PTA stained for micro-CT scanning, and of the possible 308 separate structures of interest (left and right MB lobes, MB calyces, ALs, Mes and Los, and the CB), we obtained images to allow full and detailed 3D reconstruction of 297 (96.4%) of these structures. In this study we used the 19 brains (17 workers and 2 males) that provided us with reconstructions of all the brain structures of interest per individual (see Supplementary Table 8).

### Image segmentation and geometric morphometric analyses

SPIERS 2.20 was run on a standard laptop computer (Samsung Series 3 Notebook 1TB HDD NP3530EC, Intel^®^ Core™ i5-3210M CPU @ 2.50 GHz, RAM 6.00 GB, Intel^®^ HD Graphics 3000, 64-bit Operating System). SPIERS is a custom software suite made up of three independent programs: SPIERS edit, SPIERS view and SPIERS align. SPIERS was chosen ahead of other potential software based on two key and advantageous features: i) financial cost – SPIERS is freely available; ii) system requirements – SPIERS has relatively low system requirements and will run on most standard desktop and laptop operating systems. Segmentation was carried out using the SPIERS edit program (see Supplementary Step-by-Step Guide).

For each sample, segmentation of five of the key composite structures of the brain (MBs; ALs; Mes, Los and the CB) was performed, with each MB being segmented as calyces and lobes separately. Each individual scan was opened in SPIERS edit in the form of an 8-bit greyscale bitmap (BMP) image stack. The sequence of slices could be viewed in either greyscale or threshold form (a black and white, binary visualization of the image) with a simple interchange between the two representations. For a single scan slice showing the brain region of interest, thresholding was performed by adjusting the *Base* and *Top* values representing the range of shades to be assigned on the grey-value scale covered where 0 = black and 255 = white. The optimum threshold being that which achieved the best ratio of white, active ‘on’ pixels comprising the structure of interest and black, inactive ‘off’ pixels for the surrounding tissues. To implement our ‘tracing method’ we outlined the following criteria for our threshold: i) it would separate all ‘background’ material from the bee head tissue, ii) it would retain the identifying features of each brain structure of interest (MBs, ALs, Mes, Los and CB), iii) and it would separate each structure of interest from the tissue directly surrounding it. This was achieved by overlaying the greyscale and threshold views, adjusting the base level until the binary image fulfilled our criteria - to effectively trace the structures of interest. For the ‘pixel intensity histogram’ method, we based the threshold point on the base value corresponding with the top of the second peak on the working image histogram. This second peak value was applied as the constant determinant of the threshold for the second method. The optimum threshold (for each structure) was independently determined for 15 slices, at 10 slice intervals, across one brain scan to account for slight changes in second peak values across slices. This interval spacing was selected to provide a robust subsample, as each structure is typically visible in 150 scan slices (this slice interval was adjusted for those smaller structures visible across fewer slices). The average of the ranges established for each of our 15 slices was taken to obtain a single range to apply and achieve an optimum threshold, across all scan slices for each individual scan (Fig. 1b).

Working in this ‘threshold view’, a central slice - of those in which the structure of interest was visible - was selected as a starting point. To filter down to the specific brain structure of interest we drew a looped spline around the object(s) to be followed across multiple slices and used a series of landmarks (nodes) to refine the loop to match the shape of the object as accurately as possible. We copied the fitted spline to the next slice (either five slices up or down from the starting slice) and adjusted it to fit the structure outline in the new slice (using the same number of nodes). Spline adjustment was performed on every fifth slice for the region in which the structure of interest was visible. We then interpolated the splines between each start and end slice over the five slice intervals, this automated the process of positioning nodes to define a curve on each intervening slice to create an accurate transition between curve shapes for every first to fifth slice in sequence. This produced a framework around the outline of the structure of interest across all relevant slices, which was then used as the defining foundation to create a ‘mask’, or in other words isolating the complete structure across all slices to be used to form a complete 3D image. The mask could then be exported as an independent object for reconstruction in SPIERS view rendering software, allowing 3D viewing and manipulation of the structure.

Following image reconstruction, we applied an automated processing feature in SPIERS view (called ‘island removal’ combined with ‘smoothing’) to reduce the amount of artefactual extraneous tissue and smooth the mask to depict a more accurate representation of the structure (Figs 2 and 3; see Supplementary Step-by-Step Guide for details). In SPIERS edit we then calculated the volume of the brain structure using a tool to calculate the number of voxels making-up each independent object (each segmented structure), which we could then multiply by the known voxel volume.

## Additional Information

**How to cite this article**: Smith, D. B. *et al.* Exploring miniature insect brains using micro-CT scanning techniques. *Sci. Rep.* **6**, 21768; doi: 10.1038/srep21768 (2016).

## Supplementary Material

Supplementary Information
