# Supplementary material for: Exploring miniature insect brains using micro-CT scanning techniques
Source: Sci Rep. 2016 Feb 24;6:21768. doi: 10.1038/srep21768 (PMC4764865; doi:10.1038/srep21768)
Supplement: Supplementary Information [file srep21768-s1.pdf]

SUPPLEMENTARY INFORMATION

Exploring miniature insect brains using micro-CT scanning techniques

Dylan B. Smith[1,2], Galina Bernhardt[3,4], Nigel E. Raine[2,5], Richie Abel[3], Dan Sykes[6], Farah Ahmed[6,] Inti Pedroso[2,7,§] and Richard J. Gill[1,2,§,*]

[1]Department of Life Sciences, Imperial College London, Silwood Park, Buckhurst Road, Ascot, Berkshire, SL5 7PY, UK.

[2] School of Biological Sciences, Royal Holloway University of London, Egham, Surrey, TW20 0EX, UK.

[3] Department of Surgery & Cancer, Imperial College London, MSk Laboratory, London, W6 8RP, UK.

[4] Mechatronics, School of Engineering, FHNW, Gründenstrasse 40, 4132 Muttenz, Switzerland.

[5] School of Environmental Sciences, University of Guelph, Guelph, Ontario, N1G 2W1, Canada.

[6] Core Research Laboratories, Natural History Museum, Cromwell Road, London, SW7 5BD, UK.

[7]Bio-Computing & Applied Genetics Division, Centre for Systems Biotechnology, Fraunhofer Chile Research Foundation, Avenida M. Sánchez Fontecilla 310, 7550296, Chile.

[§] Equal contribution

* Email for correspondence: r.gill@imperial.ac.uk

SUPPLEMENTARY RESULTS

For one of the 19 individuals, we found an apparent deformation in the right mushroom body (MB) lobe: this was both smaller in volume and unusual in shape. Given our typically high precision when scanning and reconstructing tissues, and the relative uniformity between right and left sides, we are confident that this was not caused by human error when segmenting the brain but

reflects true tissue deformation. Indeed this was also the brain that showed the highest percentage difference in volume calculation using the two thresholding methods.

Whilst 19 of the 28 worker brains that were scanned had all structures of interest appropriately stained, the remaining nine worker brains had some structures that did not fully stain (n = 21 separate structures in total over all nine brains; see Supplementary Table 7). We found that 15 of the 21 incompletely stained structures were the mushroom bodies (either the MB lobes or calyces). Given that mushroom bodies are the structures furthest away from the anterior sliced opening in the head-case this could suggest that the reason for poor staining could have been because we did not leave enough time for the stain to penetrate and perfuse throughout the brain. We found that the nine bees that were incompletely stained were significantly larger than the workers that had fully stained brains (median (IQR): 5.09 (4.73-5.38) vs 4.57 (4.22-4.94) mm; Mann Whitney: $U = 18$, $n_1 = 9$, $n_2 = 19$, $p = 0.044$). It therefore may be advisable to stain the brain for an extra day (up to 8 days staining) if the individual in question is comparatively large.

We compared the different brain structures against each other in a pair-wise manner to examine whether the ratios between pairs of each structure remained consistent with increasing body size (in this analysis we considered the calyces and lobes independently rather than mushroom body as a whole). We did not find any significant change after correcting for multiple testing for the 15 paired comparisons (p-value <0.033: see Supplementary Figure 6).

Supplementary Videos

To access the videos please use the following Figshare links:

Supplementary video 1: shows a scrolling view through the brain with the raw CT image slices.

https://figshare.com/s/0a22612f83150e70632c

Supplementary video 2: an exploration of the brain.

https://figshare.com/s/ed9f8e7d3e92f2335934

Supplementary Figures & Tables

Supplementary Figure 1: A) Lateral view showing the cutting planes to remove the bee's head from the body (decapitation: red dotted line) and to open the head case to expose the brain for staining (blue dotted line). B) Dorsal view showing the standardised thorax width measurement (black dotted line) across the widest parts of the tegulas (insertion points for the wings on each side of the thorax). The mean thorax width across the 19 workers was 4.63 mm (range = 3.61 – 5.61).

A

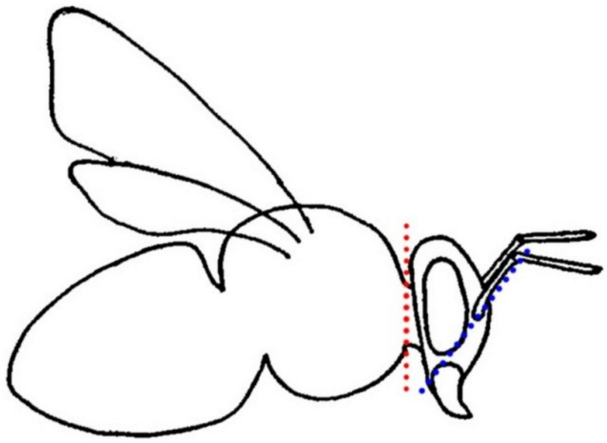

B

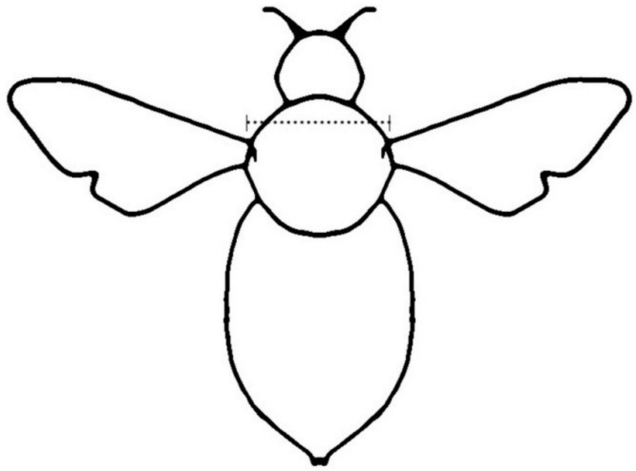

Supplementary Figure 2. Scanned images from the stain optimization test. Each scan slice shows the degree of staining for each day (days 1, 3, 6, 7, 8 and 9) and stain (Uranyl acetate (UA), Iodine (I) and phosphotungstic acid (PTA)). Progressive staining across days is illustrated by the receding dark area revealing more brain tissue over the staining period. Each stain perfused at different rates reaching their optimum at days 1 and 7 for Iodine and PTA respectively, with UA not fully penetrating the whole brain after 9 days (showing a very slow perfusion rate). Each image shows a slice that best represents the level of perfusion through the brain whilst attempting to show all the brain structures of interest.

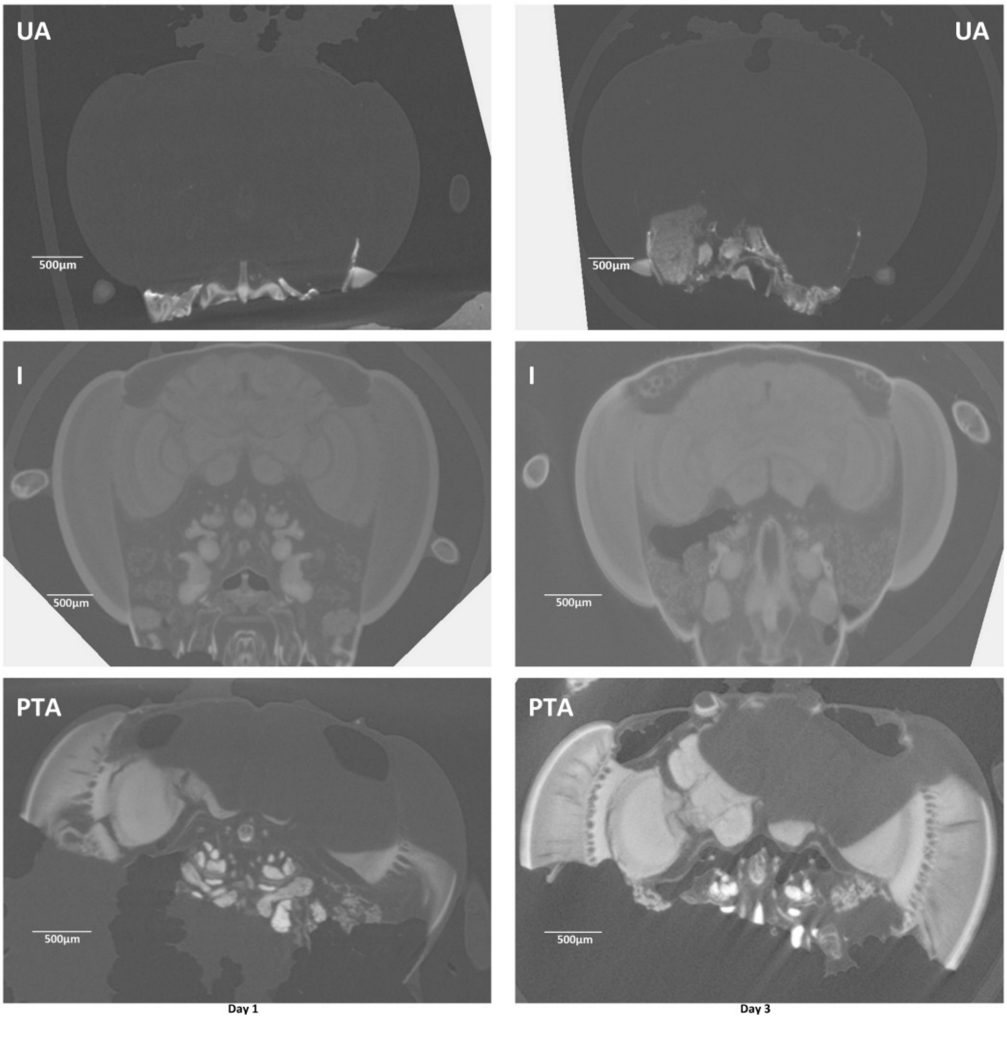

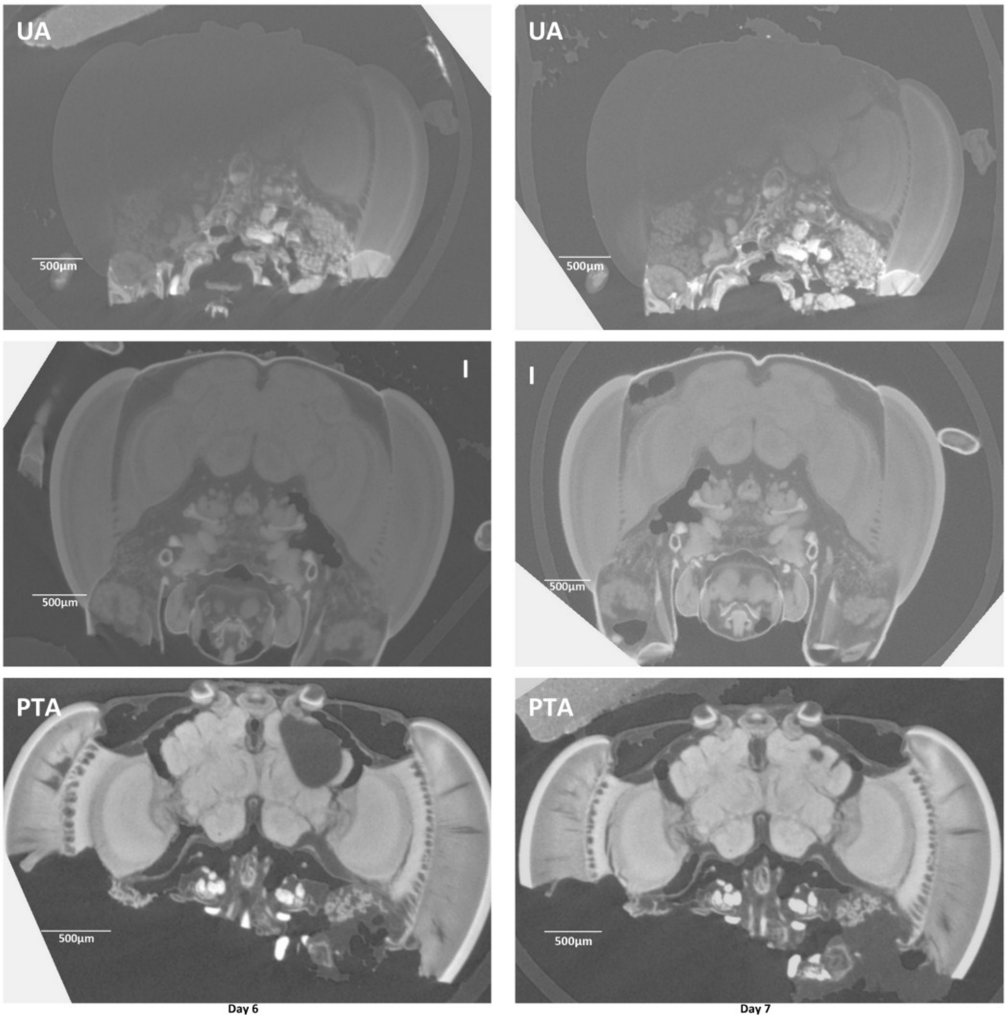

Day 6

Day 7

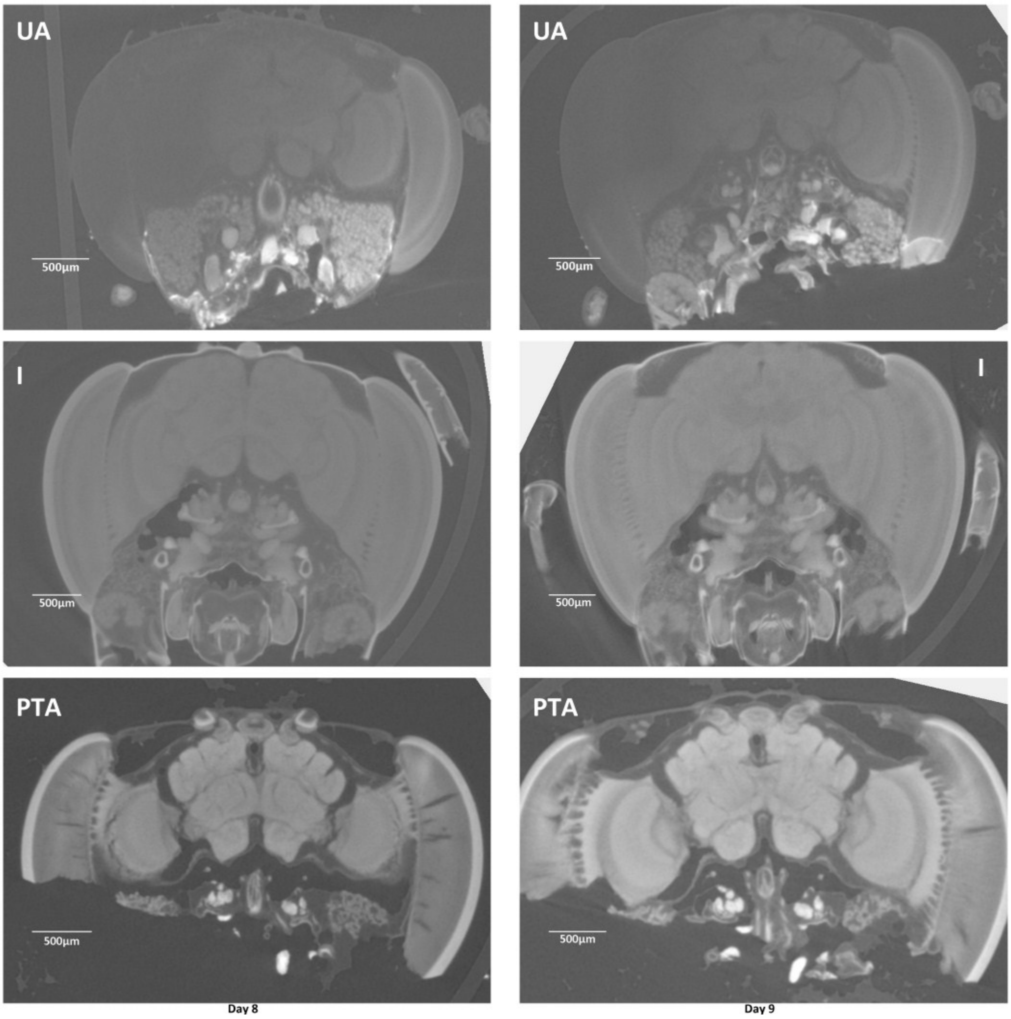
UA
500µm
UA
500µm
I
500µm
I
500µm
PTA
500µm
PTA
500µm
Day 8
Day 9

Supplementary Figure 3. Day 7 scan images and contrast histograms. Slice images for each stain UA, I and PTA are aligned to compare and illustrate the level of contrast enhancement when adjusted for optimum window width / level (UA = 142 / 164; I = 130 / 173; PTA = 172 / 149), with the associated histograms for each stain showing the distribution of the pixel values on the greyscale (0-255). Each slice is representative of the staining seen throughout the brain when comparing all slices. A wider pixel distribution across the greyscale indicates greater tissue differentiation for identifying and segmenting individual structures and regions. Comparison of histograms shows that PTA stain gives the best contrast enhancement.

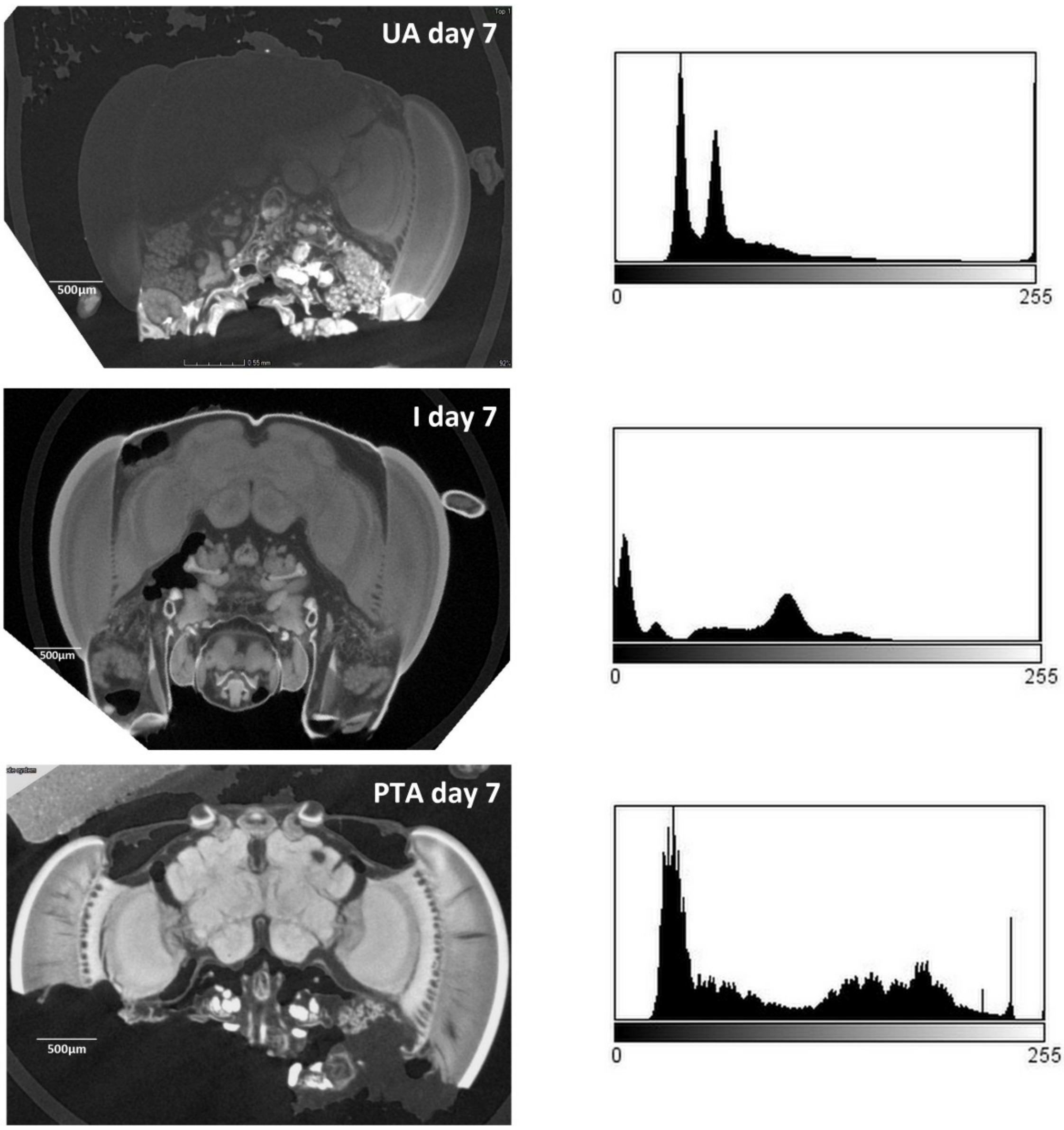

Supplementary Figure 4. Comparison of scan images and contrast histograms at the days showing the greatest contrast enhancement when adjusted for optimum window width / level (UA = 142 / 164; I = 130 / 173; PTA = 172 / 149).

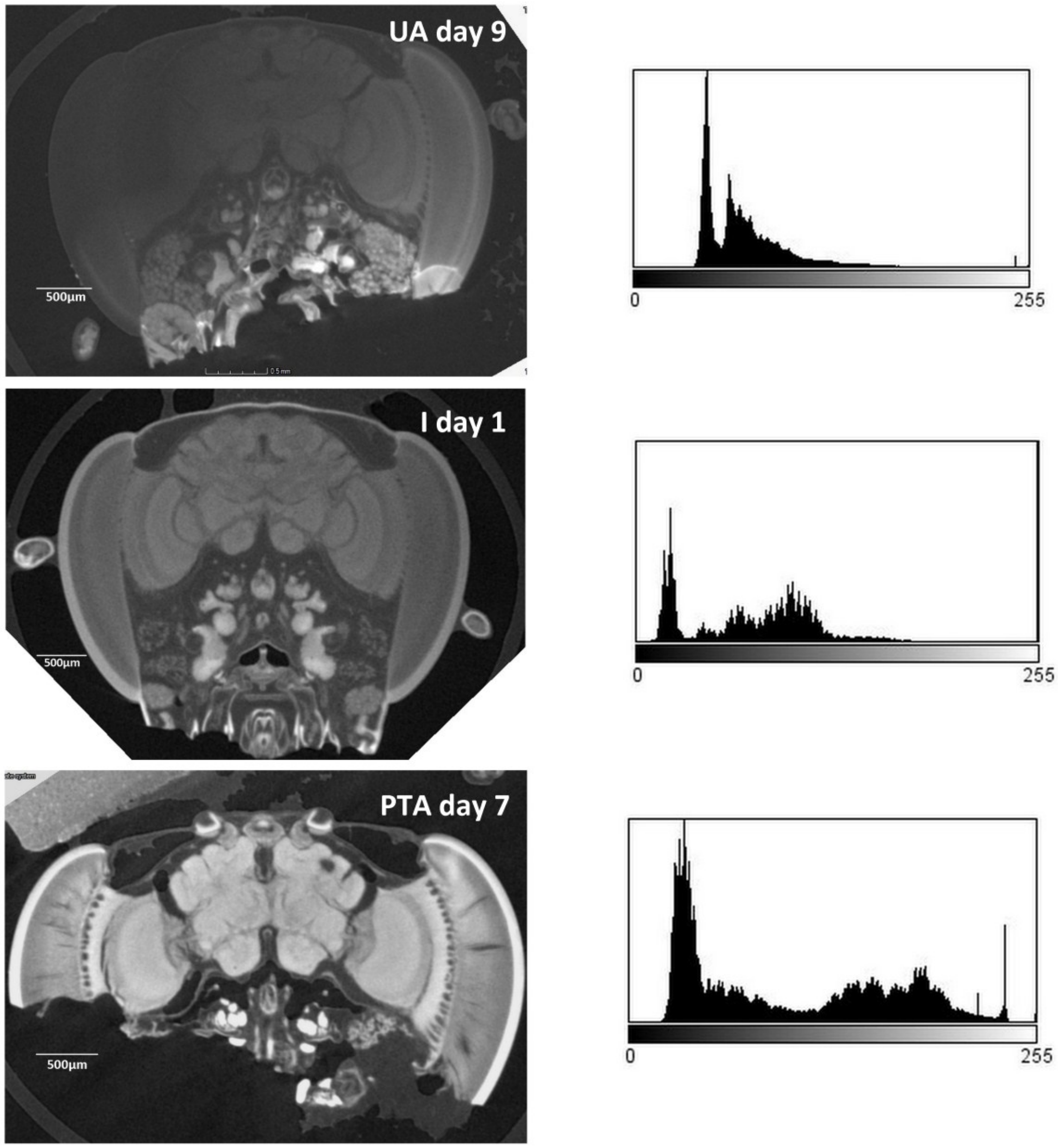

Supplementary Figure 5. Metris X-Tek HMX ST 225 Micro-CT Scanner at the *Imaging and Analysis Centre*, London Natural History Museum (image courtesy of Dan Sykes and Farah Ahmed).

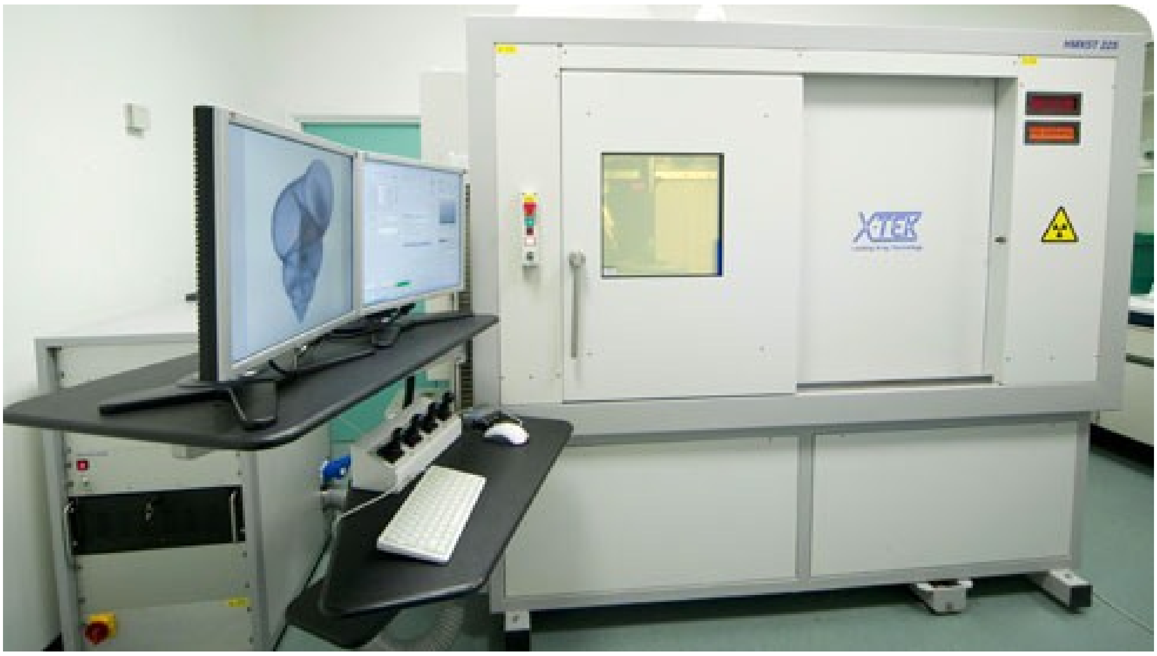

Supplementary Figure 6. Comparison of the optimum base levels used with the manual tracing method (open circles) versus the histogram pixel intensity method (filled circles). As calyces are complex structures there is more likelihood of finding differences in base level using each method, whereas the simpler structure of medullas results in lower disparity.

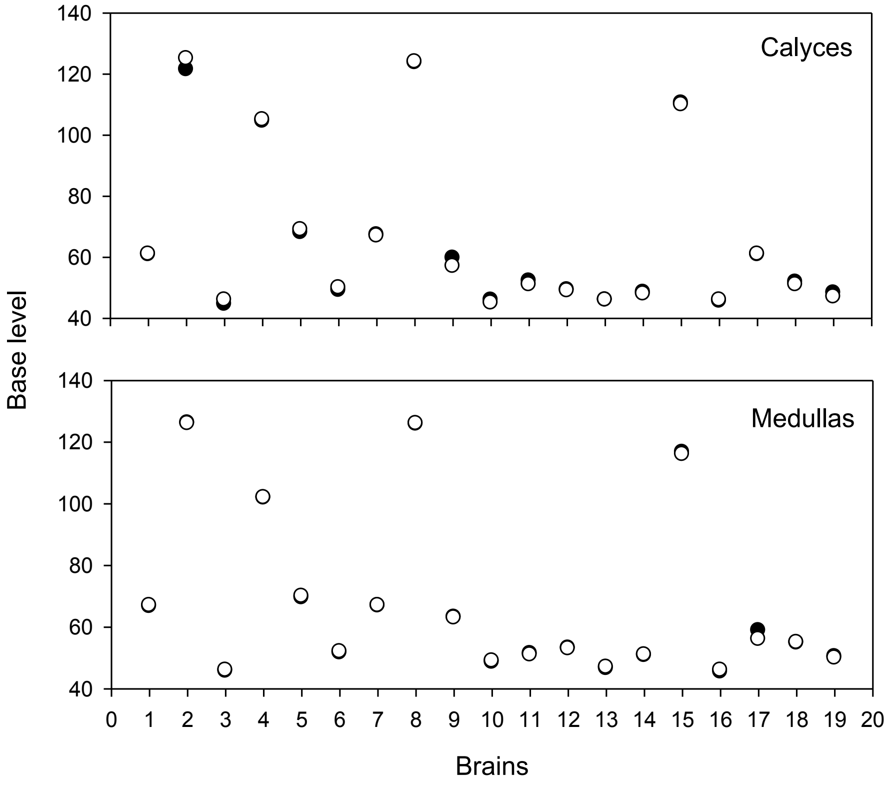

Supplementary Figure 7: Correlations between calculated volumes (mm^3) using the manual tracing method of paired structures found on the left (x-axis) and right (y-axis) sides of the brain (n = 19). Fitted linear regression lines are plotted with r^2 values and slope gradients shown.

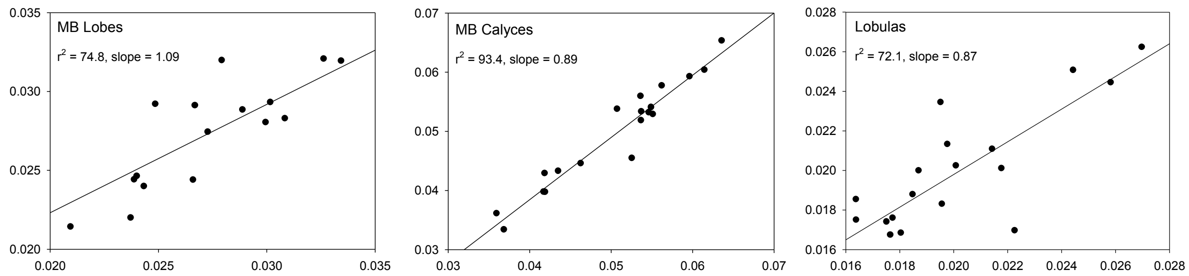

Supplementary Figure 8. Correlations between the calculated volumes (mm^3) using the histogram intensity method of paired structures found on the left (x-axis) and right (y-axis) sides of the brain (n = 19). Fitted linear regression lines are plotted with r^2 values and slope gradients shown.

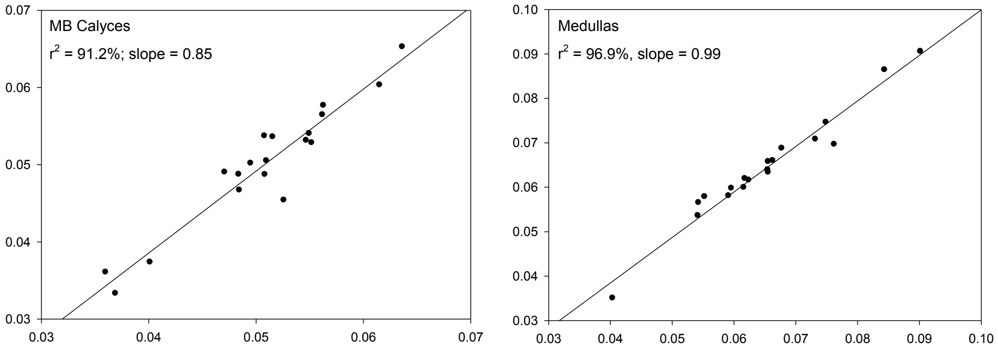

Supplementary Figure 9. Body size and brain structure relationship. Comparison between body size (determined here by thorax width/ mm) and volume (mm^3) of brain structures of interest calculated using the manual tracing method: mushroom bodies (MBs), central body and lobulas (n = 19 workers; volumes of paired structures were combined to provide a total volume for this analysis). Fitted linear regression lines are plotted with 95% confidence limits (dashed line), and r^2 values and slope gradients are shown.

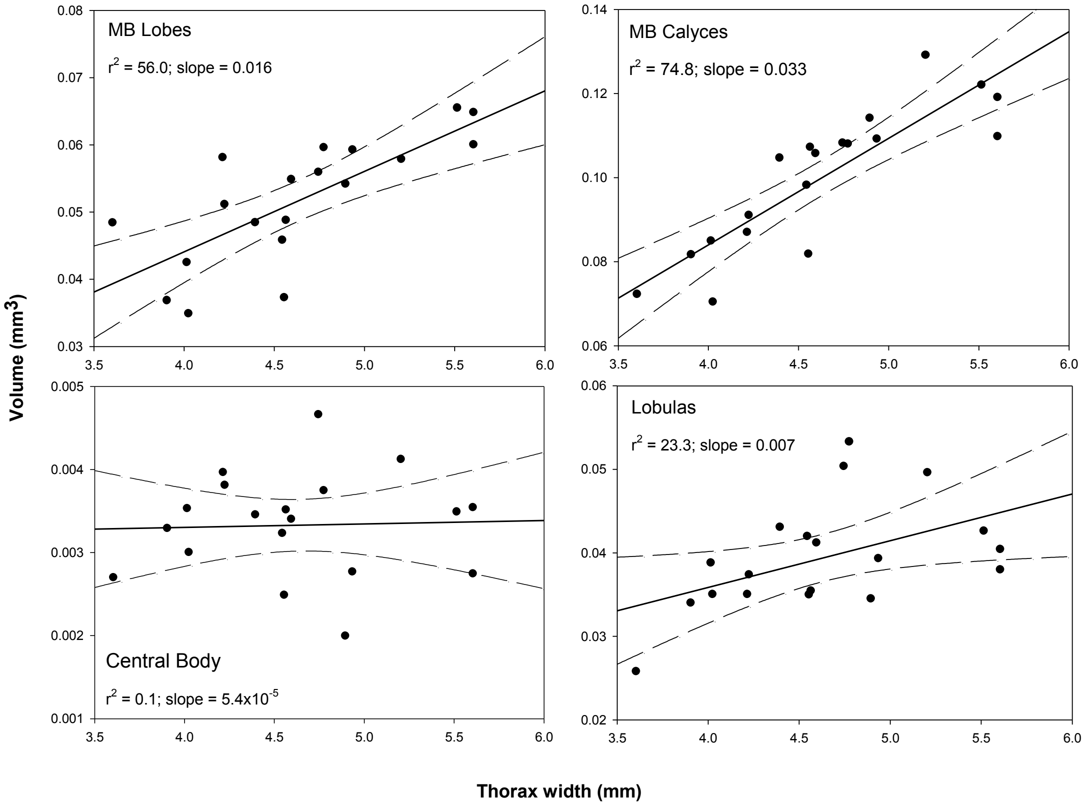

Supplementary Figure 10. Taking a pairwise approach, the associated volumes (mm^3) of each brain structure (calculated using the manual tracing method) were compared to provide a comparative size ratio across all 19 individuals. These ratios were then plotted against the associated individual body size (thorax width /mm) to explore whether there were relative differences between structure sizes in different sized bees. Fitted linear regression lines are plotted with 95% confidence limits (dashed line) along with the statistical output from the regression analysis with p-values falling under the 0.05 significance value highlighted in bold. For the mushroom body (MB) we explored the relationship when the lobes and calyces were combined (Whole MB) as well as independently.

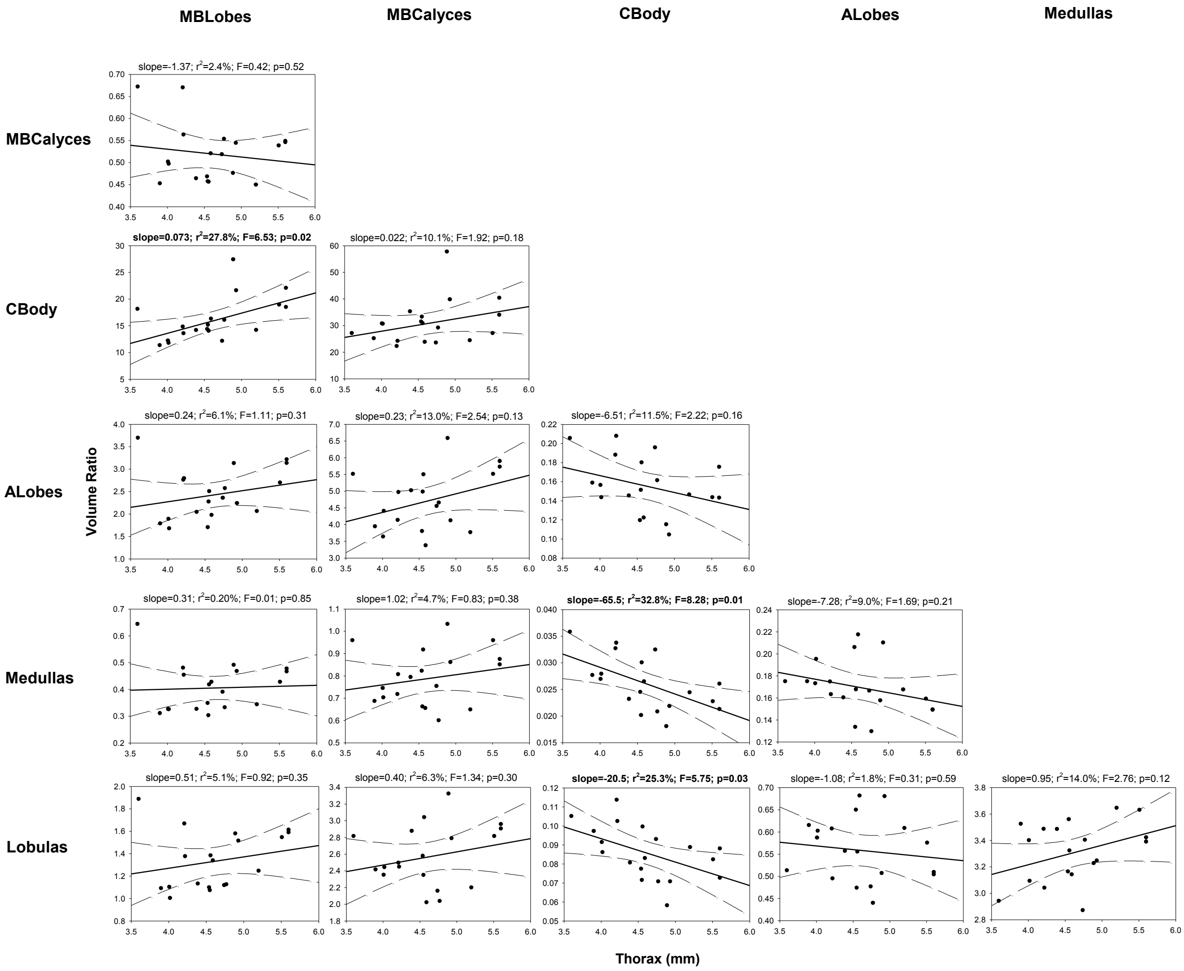

Supplementary Table 1. Studies of bee brain volumes using a variety of traditional histological techniques for sample preparation and staining.

Study	Species	Sample Preparation	Stain	Slice Thickness	Volume Calculation
1	*Bombus impatiens* and *Apis mellifera*	Decapitated, head capsule cut open frontally, brain dissected out under fixative (4% formaldehyde and 0.1% picric acid in phosphate buffer, pH 6.8) and fixed for 3 hours. Rinsed in four repeated changes of buffer, stained in 1% aqueous osmium tetroxide solution for 2 hours at 4°C and for one additional hour at room temperature. Next, brains rinsed in water for 4 hours, dehydrated in acidified 2,2-dimethoxypropane [Thorpe and Harvey, 1979], plastic-embedded (Fluka, Durcupan) and polymerized at 65°C. Sectioned on a sliding microtome.	1% aqueous osmium tetroxide	10-20µm	Area measurements multiplied by the section thickness and number of sections. Every second section (20-um sections) or every third section (10-um sections) was traced and measured
2	*Bombus occidentalis*	Dissected from head capsule, fixed in 4% phosphate-buffered formaldehyde for 2 hours, then rinsed in phosphate buffer. Brains then stained in the dark using 1% osmium-tetroxide for 2 hours at 4°C and an additional 30 minutes at room temperature. After repeated rinses with distilled water, dehydrated brains with 50% ethanol (10 min), acidified 2,2-dimethoxypropane [10 min; Thorpe and Harvey, 1979] and acetone (10 minutes). Next, brains plastic-embedded (Spurr's low viscosity medium, RT 14300 Electron Microscopy Sciences) and sectioned on a sliding microtome.	1% osmium-tetroxide	10-20µm	Multiplying the area of each region by the section thickness. Outlined and measured every second (20µm) or every third (10µm) section.

3	*Apis mellifera*	Heads embedded in wax, opened frontally and brains dissected under 50% ethanol, then transferred to water and block-stained in toluidine blue (1% toluidine blue in 1% aqueous borax solution) overnight at room temperature on a rotator. The staining then differentiated in water for 3–6 hours, transferred to 50% ethanol and then dehydrated in acidified 2,2-dimethoxypropane (Thorpe & Harvey, 1979), embedded in Spurr's low viscosity embedding medium (Electron Microscopy Science; Hatfield, PA) and polymerized at 70°C. Brains sectioned on a sliding microtome.	1% toluidine blue in 1% aqueous borax solution	20µm	Area measurements multiplied by section thickness. Every second section was measured, probing the brains at 40µm intervals.
4	*Bombus impatiens*	Decapitated and removed mandibles and part of each eye to allow fixative to penetrate. Brains fixed within head capsule in 4% formaldehyde in cacodylate buffer (pH 6.8) overnight on a rotator. After fixation, brains rinsed with and stored in cacodylate buffer at 4°C until dissection. Brains dissected from the head capsule and stained in the dark using 1% aqueous osmium tetroxide for 2 hours on ice, then an additional 30 minutes at room temperature. After rinsing with distilled water, brains were dehydrated using 50% ethanol, acidified 2,2-dimethoxypropane [Thorpe and Harvey, 1979], and acetone for 10 minutes each. Next, brains were plastic-embedded in Spurr's low-viscosity medium (RT 14300; Electron Microscopy Sciences). Blocks polymerized at 65°C for 12 hours, sectioned on a sliding microtome.	1% aqueous osmium tetroxide	10 or 15µm	Multiplying area by the section thickness. Every other section (15-µm section thickness) or every third section (10-µm section thickness) was measured.
5	*Melipona quadrifasciata anthidioides*	Brains were dissected in insect physiological solution; the tissues then fixed in 4% paraformaldehyde in 0.1 M phosphate buffer (pH 7.4) for 24 hours. Fixed samples then rinsed in 0.1 M phosphate-buffered saline (PBS), dehydrated in an increasing series of ethanol concentrations (70–100 %), and embedded in JB4 historesin (EMS) according to the manufacturer's instructions. Brains were subjected to serial sectioning using a glass knife on an automatic microtome.	Hematoxylin and eosin	7µm	Measurements performed at each of six-section intervals, following Cavalieri's method.

Supplementary Table 2. Studies investigating correlations between insect brain morphology, behaviour and performance.

Study	Species	Brain Structure(s)	Findings
6	*Apis mellifera*	Mushroom bodies	Mushroom body volume increased with foraging experience
7	*Apis mellifera*	Mushroom body calyces	Experience dependent volume changes of calyces subcompartments (lip and collar) in foragers compared to nurses and newly emerged bees
8	*Apis mellifera*	Antennal lobes	Behaviour and task dependent volumetric changes of antennal lobes
9	*Apis mellifera*	Antennal lobes	Olfactory learning performance increased with increased antennal lobe volume and an activity dependent volume increase in antennal lobes
10	*Apis mellifera*	Mushroom bodies	Mushroom body exhibited experience-expectant volume increase independent of light stimulus and social interaction
11	*Messor pergandei* and *Pogonomyrmex rugosus*	Medulla, lobula and mushroom bodies	Decreasing phototaxis correlated with decreased medulla volume
12	*Osmia lignaria*	Mushroom bodies	Mushroom body volume increased with foraging experience
3	*Apis mellifera* European and Africanized Honeybee	Mushroom bodies and lobula	Mushroom body volume increased with olfactory learning performance but lobula volume correlated negatively with olfactory learning performance
2	*Bombus occidentalis*	Mushroom body, MB calyces and antennal lobes	Foraging experience did not correlate with mushroom body total volume or volume of calyces but did positively correlate with volume of the medial calyx. Found no experience-dependent volume increase of the antennal lobes
4	*Bombus impatiens*	Mushroom bodies and antennal lobes	Found that bee deprived of visual stimuli for 7 days had increased mushroom body and antennal lobe volume than bees exposed to visual stimuli

Supplementary Table 3. Functions assigned to the insect brain structures we focus on in this study.

**Structure**	**Function**	**References**
Mushroom Bodies	Associated with higher cognition and learning with the processing of multimodal sensory information. They possess distinct subcompartments of functional specialisation.	[7] [13] [14] [15] [16]
Antennal Lobes	The principle olfactory centre associated with processing of chemical stimuli.	[17], [18]
Medullas	Processing of visual information	[19] [20]
Lobulas	Processing of visual information	[19] [20] [21]
Central Body (Fan Shaped Body)	Considered to be involved in locomotion and orientation	[22] [23] [24]

Supplementary Table 4. Comparison of the base level (*bl*) threshold value and resulting volumetric calculations based on the manual tracing and histogram pixel intensity methods, showing the percentage difference between the two for the MB calyces and medullas. The complex structure of the calyces shows that using alternative methods can result in different volume calculations for nine of the 19 brains because of *bl* differences. For the simpler structure of the medulla, however, only two of the 19 brains showed different calculated volumes. The brain number corresponds to the x axis number in Figure S6. Note that as previously mentioned the MB calyces of bee C3W7 was observed to be malformed which made tissue differentiation for segmentation difficult and likely explains why there is a big disparity when using the two methods.

**Bee**	**Brain**	**MB Calyces**					**Medullas**				
		**manual**		**histogram**		**% diff.**	**manual**		**histogram**		**% diff.**
		bl	***vol. / mm3***	***bl***	***vol. / mm3***		***bl***	***vol. / mm3***	***bl***	***vol. / mm3***	
C3W41	1	46	0.129	46	0.129	0	46	0.171	46	0.171	0
C2G3	2	45	0.085	46	0.096	11.8	46	0.131	46	0.131	0
C2G8	3	124	0.098	124	0.098	0	126	0.132	126	0.132	0
C2G27	4	52	0.105	51	0.100	6.0	51	0.129	51	0.129	0
C3W5	5	105	0.070	105	0.070	0	102	0.108	102	0.108	0
C3W25	6	67	0.104	67	0.104	0	67	0.149	67	0.149	0
C12G28	7	61	0.122	61	0.122	0	59	0.154	56	0.146	5.6
C12G13	8	61	0.072	61	0.072	0	67	0.075	67	0.075	0
C12G12	9	48	0.114	48	0.114	0	51	0.111	51	0.111	0
C11R21	10	60	0.082	57	0.077	5.3	63	0.124	63	0.124	0
C6W11	11	46	0.107	45	0.101	5.5	49	0.117	49	0.117	0
C6W13	12	49	0.091	50	0.095	4.5	52	0.113	52	0.113	0
C11R13	13	52	0.110	51	0.105	4.2	55	0.129	55	0.129	0
C3W1	14	46	0.108	46	0.108	0	47	0.181	47	0.181	0
C2G29	15	110	0.109	110	0.109	0	117	0.127	116	0.124	3.0
C3W7	16	121	0.081	125	0.100	18.3	126	0.119	126	0.119	0
C6W52	17	48	0.119	47	0.113	5.5	50	0.136	50	0.136	0
C12G1	18	49	0.108	49	0.108	0	53	0.144	53	0.144	0
C12G11	19	68	0.087	69	0.097	10.6	70	0.121	70	0.121	0

Supplementary Table 5. Repeated segmentation and volumetric calculations of the right and left medullas from a single brain using the manual tracing method. The top table shows the basic statistics of the four repeated measures per structure, with the 'test re-test repeatability' calculated by multiplying the standard deviation by 1.96. The bottom table shows a pairwise comparison for each repeated volume estimate per structure and takes the mean and median percentage differences over all six comparisons to give an inaccuracy value.

Repeated measure	Vol. Right Medulla	Vol. Left Medulla
**1**	0.0749778	0.0744753
**2**	0.0750145	0.0757387
**3**	0.0733002	0.0749277
**4**	0.0746532	0.0754171
**Mean**	0.0745	0.0751
**Standard deviation**	0.0008	0.0006
**Standard error of mean**	0.0004	0.0003
**Test re-test repeatability score**	0.0016	0.0011
**Co-efficient of variation (%)**	1.0838	0.7378

**Pair-wise comparison of % differences between each repeated measure**				
Medulla 1				
	0.0749778	0.0750145	0.0733002	0.0746532
0.0749778	x			
0.0750145	0.048881778	x		
0.0733002	2.237461569	2.285226288	x	
0.0746532	0.433011087	0.481657423	1.845748393	x
**Mean % diff**	1.2220	(mean of all six pairwise comparisons)		
**Median % diff**	1.1637	(median of all six pairwise comparisons)		
Medulla 2				
	0.0744753	0.0757387	0.0749277	0.0754171
0.0744753	x	x	x	x
0.0757387	1.6964	x	x	x
0.0749277	0.6075	1.0707	x	x
0.0754171	1.2645	0.4246	0.6531	x
**Mean % diff**	0.9528	(mean of all six pairwise comparisons)		
**Median % diff**	0.8619	(median of all six pairwise comparisons)		

Supplementary Table 6. Statistical outputs from paired t-tests comparing the calculated volumes of each brain structure from the right and left side of the brain. The table shows the mean values (± s.e.m.) for each of the paired structures (excluding the central body as this is not a paired structure) as well as the total volumes when both of paired structures are combined. All volumes were calculated using the manual tracing method, except those values underlined calculated using the histogram intensity method for comparison.

**Structure**	**Right side (mm3)**		**Left side (mm3)**		**n**	**t**	**p**	**Total**	
	**mean**	**s.e.**	**mean**	**s.e.**				**mean**	**s.e.**
Whole MBs	0.07606	0.00288	0.07563	0.00287	38	0.48	0.637	0.15169	0.00567
MB Lobes	0.02554	0.00123	0.02612	0.00097	38	-0.84	0.413	0.05166	0.00211
MB Calyces	0.05052	0.00188	0.04951	0.00203	38	1.97	0.065	0.10002	0.00386
<u>MB Calyces</u>	<u>0.05095</u>	<u>0.00168</u>	<u>0.05011</u>	<u>0.00186</u>	<u>38</u>	<u>1.42</u>	<u>0.173</u>	<u>0.10100</u>	<u>0.00347</u>
Antennal Lobes	0.01088	0.00044	0.01095	0.00051	38	-0.27	0.790	0.02183	0.00092
Medullas	0.06558	0.00268	0.06460	0.00272	38	1.54	0.142	0.13019	0.00540
<u>Medullas</u>	<u>0.06516</u>	<u>0.00261</u>	<u>0.06437</u>	<u>0.00270</u>	<u>38</u>	<u>1.53</u>	<u>0.144</u>	<u>0.12956</u>	<u>0.00532</u>
Lobulas	0.01978	0.00078	0.01962	0.00074	38	0.62	0.543	0.03941	0.00150
Central Body	-	-	-	-	19	-	-	0.00333	0.00014

Supplementary Table 7: Volumetric calculations using the manual tracing method for each structure from our study compared with those found in two other high quality histological studies (Mares *et al.* 2005; Jones *et al.* 2013). Volumes for each paired structure were summed to provide a total volume per individual, and volumes presented have been standardised relative to the respective individual's whole brain volume (no. individuals: our study = 19, Mares *et al.* = 46, Jones *et al.* = 10). Both studies by Mares *et al*. (2005) and Jones *et al.* (2013) were on *Bombus impatiens* (full worker body length = 8.5-16mm) with our study on *B. terrestris* (full worker body length = 11-17mm). Considering values at four decimal places we compared our volumetric calculations per brain structure to those found in the two comparative studies and show the absolute different in value by subtracting the comparative studies from ours. We additionally present this calculated value as a percentage reduction (% difference) from the volumes calculated from Mares *et al.* (2005) or Jones *et al.* (2013).

	Our Study		Mares 2005		Jones 2013*		**Our study minus Mares**		**Our study minus Jones**	
	**mean**	**s.e.m**	**mean**	**s.e.m**	**mean**	**s.e.m**	**value difference**	**% difference**	**value difference**	**% difference**
Whole MBs	0.118660	0.002962	0.24	0.02	0.1709	-	-0.1213	-50.6	-0.0522	-30.6
MB Lobes	0.040434	0.001244	0.05	0.01	0.0670	0.0048	-0.0096	-19.1	-0.0266	-39.7
MB Calyces	0.078226	0.002068	0.19	0.02	0.1039	0.0062	-0.1118	-58.8	-0.0257	-24.7
Antennal Lobes	0.017532	0.000838	0.04	0.01	0.033	0.0034	-0.0225	-56.2	-0.0155	-46.9
Medullas	0.102012	0.003386	0.17	0.02	0.1815	0.0088	-0.0680	-40.0	-0.0795	-43.8
Lobulas	0.031373	0.001177	0.05	0.01	0.0535	0.002	-0.0186	-37.3	-0.0221	-41.4
Central Body	0.002628	0.000118	0.003	0.001	0.0033	0.0003	-0.0004	-12.4	-0.0007	-20.4

Supplementary Table 8. Table showing which structures were successfully (x) and unsuccessfully (-) stained and segmented from the 28 bee brains scanned the five sampled colonies. Any brains that had at least one structure unsuccessfully stained were excluded from final volumetric analyses. Table also shows the number of CT scan slices that were obtained from each scan per brain.

Colony	Worker ID	No. Scan Slices	MBLobes		MBCalyces		CB	AL		Medulla		Lobulla		Used in Volumetric analysis
			L	R	L	R		L	R	L	R	L	R	
C11	R1	448	x	-	x	-	x	x	x	x	x	x	x	N
C11	R13	336	x	x	x	x	x	x	x	x	x	x	x	Y
C11	R19	258	-	x	-	x	x	-	-	x	x	x	x	N
C11	R21	361	x	x	x	x	x	x	x	x	x	x	x	Y
C11	R8	364	-	x	-	x	x	x	x	x	x	x	x	N
C11	R9	314	-	x	-	x	-	x	x	x	x	x	x	N
C12	G1	323	x	x	x	x	x	x	x	x	x	x	x	Y
C12	G11	428	x	x	x	x	x	x	x	x	x	x	x	Y
C12	G12	335	x	x	x	x	x	x	x	x	x	x	x	Y
C12	G13	294	x	x	x	x	x	x	x	x	x	x	x	Y
C12	G16	362	x	x	x	-	x	x	x	x	x	x	x	N
C12	G27	420	-	-	-	-	-	x	x	x	x	x	x	N
C12	G28	343	x	x	x	x	x	x	x	x	x	x	x	Y
C2	G27	339	x	x	x	x	x	x	x	x	x	x	x	Y
C2	G29	454	x	x	x	x	x	x	x	x	x	x	x	Y
C2	G3	366	x	x	x	x	x	x	x	x	x	x	x	Y
C2	G8	580	x	x	x	x	x	x	x	x	x	x	x	Y
C3	W1	434	x	x	x	x	x	x	x	x	x	x	x	Y
C3	W25	319	x	x	x	x	x	x	x	x	x	x	x	Y
C3	W41	372	x	x	x	x	x	x	x	x	x	x	x	Y
C3	W5	420	x	x	x	x	x	x	x	x	x	x	x	Y
C3	W6	241	x	x	-	x	x	x	x	x	x	x	x	N
C3	W7	340	x	x	x	x	x	x	x	x	x	x	x	Y
C6	W1	353	x	x	-	x	x	x	x	x	x	x	x	N
C6	W11	469	x	x	x	x	x	x	x	x	x	x	x	Y
C6	W12	323	x	x	x	x	x	-	-	x	x	x	x	N
C6	W13	296	x	x	x	x	x	x	x	x	x	x	x	Y
C6	W52	353	x	x	x	x	x	x	x	x	x	x	x	Y

SUPPLEMENTARY REFERENCES

1 Mares, S., Ash, L. & Gronenberg, W. Brain allometry in bumblebee and honey bee workers. *Brain, Behavior and Evolution* **66**, 50-61, doi:10.1159/000085047 (2005).

2 Riveros, A. J. & Gronenberg, W. Brain allometry and neural plasticity in the bumblebee *Bombus occidentalis*. *Brain, Behavior and Evolution* **75**, 138-148 (2010).

3 Gronenberg, W. & Couvillon, M. J. Brain composition and olfactory learning in honey bees. *Neurobiology of Learning and Memory* **93**, 435-443, doi:10.1016/j.nlm.2010.01.001 (2010).

4 Jones, B. M., Leonard, A. S., Papaj, D. R. & Gronenberg, W. Plasticity of the Worker Bumblebee brain in relation to age and rearing environment. *Brain, Behavior and Evolution* **82**, 250-261 (2013).

5 Tomé, H., Rosi-Denadai, C., Pimenta, J., Guedes, R. & Martins, G. Age-mediated and environmentally mediated brain and behavior plasticity in the stingless bee *Melipona quadrifasciata anthidioides*. *Apidologie* **45**, 557-567, doi:10.1007/s13592-014-0272-7 (2014).

6 Withers, G. S., Fahrbach, S. E. & Robinson, G. E. Selective neuroanatomical plasticity and division of labour in the honeybee. *Nature* **364**, 238-240 (1993).

7 Durst, C., Eichmüller, S. & Menzel, R. Development and experience lead to increased volume of subcompartments of the honeybee mushroom body. *Behavioral and Neural Biology* **62**, 259-263, doi: 10.1016/S0163-1047(05)80025-1 (1994).

8 Winnington, A. P., Napper, R. M. & Mercer, A. R. Structural plasticity of identified glomeruli in the antennal lobes of the adult worker honey bee. *Journal of Comparative Neurology* **365**, 479-490, doi:10.1002/(SICI)1096-9861(19960212)365:3<479::AID-CNE10>3.0.CO;2-M (1996).

9 Sigg, D., Thompson, C. M. & Mercer, A. R. Activity-dependent changes to the brain and behavior of the honey bee, *Apis mellifera* (L.). *Journal of Neuroscience* **17**, 7148-7156 (1997).

10 Fahrbach, S. E., Moore, D., Capaldi, E. A., Farris, S. M. & Robinson, G. E. Experience-expectant plasticity in the mushroom bodies of the honeybee. *Learning & Memory* **5**, 115-123 (1998).

11 Julian, G. E. G., W. Reduction of brain volume correlates with behavioral changes in queen ants. *Brain, Behaviour and Evolution* **60**, 152-164 (2002).

12 Withers, G. S., Day, N. F., Talbot, E. F., Dobson, H. E. M. & Wallace, C. S. Experience-dependent plasticity in the mushroom bodies of the solitary bee *Osmia lignaria* (Megachilidae). *Developmental Neurobiology* **68**, 73-82, doi:10.1002/dneu.20574 (2008).

13 Homberg, U. & Ereber, J. Response characteristics and identification of extrinsic mushroom body neurons of the bee. *Zeitschrift für Naturforschung* **34**, 612–615 (1979).

14 Erber, J., Homberg, U. & Gronenberg, W. Functional roles of the mushroom bodies in insects. *Arthropod brain: its evolution, development, structure and functions. Wiley, New York*, 485-511 (1987).

15 Mobbs, P. G. The brain of the honeybee *Apis Mellifera*. I. The connections and spatial organization of the mushroom bodies. *Philosophical Transactions of the Royal Society-B, Biological Sciences* **298**, 309-354, doi:10.2307/2395972 (1982).

16 Fahrbach, S. E. Structure of the mushroom bodies of the insect brain. *Annual Review of Entomology* **51**, 209-232, doi:10.1146/annurev.ento.51.110104.150954 (2006).

17 Hansson, B. S. & Anton, S. Function and morphology of the antennal lobe: new developments. *Annual Review of Entomology* **45**, 203-231, doi:10.1146/annurev.ento.45.1.203 (2000).

18 Homberg, U., Christensen, T. A. & Hildebrand, J. G. Structure and function of the deutocerebrum in insects. *Annu Review of Entomology* **34**, 477-501, doi:10.1146/annurev.en.34.010189.002401 (1989).

19 Paulk, A. C., Dacks, A. M. & Gronenberg, W. Color processing in the medulla of the bumblebee (Apidae: *Bombus impatiens*). *Journal of Comparative Neurology* **513**, 441-456, doi:10.1002/cne.21993 (2009).
20 Paulk, A. C., Dacks, A. M., Phillips-Portillo, J., Fellous, J.-M. & Gronenberg, W. Visual processing in the central bee brain. *Journal of Neuroscience* **29**, 9987-9999, doi:10.1523/JNEUROSCI.1325-09.2009 (2009).
21 Paulk, A. C., Phillips-Portillo, J., Dacks, A. M., Fellous, J.-M. & Gronenberg, W. The processing of color, motion, and stimulus timing are anatomically segregated in the bumblebee brain. *Journal of Neuroscience* **28**, 6319-6332, doi:10.1523/jneurosci.1196-08.2008 (2008).
22 Strauss, R. & Heisenberg, M. A higher control center of locomotor behavior in the *Drosophila* brain. *Journal of Neuroscience* **13**, 1852-1861 (1993).
23 Strauss, R. The central complex and the genetic dissection of locomotor behaviour. *Current Opinion in Neurobiology* **12**, 633-638, doi: 10.1016/S0959-4388(02)00385-9 (2002).
24 Li, W. *et al.* Morphological characterization of single fan-shaped body neurons in *Drosophila melanogaster*. *Cell and Tissue Research* **336**, 509-519, doi:10.1007/s00441-009-0781-2 (2009).

Step-by-Step Guide to using SPIERS for segmentation of soft tissue structures from micro-CT scan images

Dylan B. Smith§, Galina Bernhardt, Nigel E. Raine, Richie Abel, Dan Sykes, Farah Ahmed, Inti Pedroso and Richard J. Gill*

§ Majority contribution to this step-by-step guide

* Email for correspondence: r.gill@imperial.ac.uk

The following step-by-step guide presents our manual threshold method and shows how a structural component of the mushroom body (MB), the calyces, can be identified and segmented from the surrounding brain soft tissue using the images gained from micro-CT scanning a bumblebee (*Bombus terrestris*) brain.

The segmentation of the MB calyces from the brain was carried out using the package *SPIERS edit* and *SPIERS view* using the following optimised protocol:

Step 1

Open an individual scan In SPIERS edit in the form of 8-bit greyscale bitmap (BMP) image series (File > New > select all images > Open). Press the Space bar to exit the primary setting of the threshold view, the image series can then be visualized as the greyscale series, using the scroll bar beneath the image panel ↑.

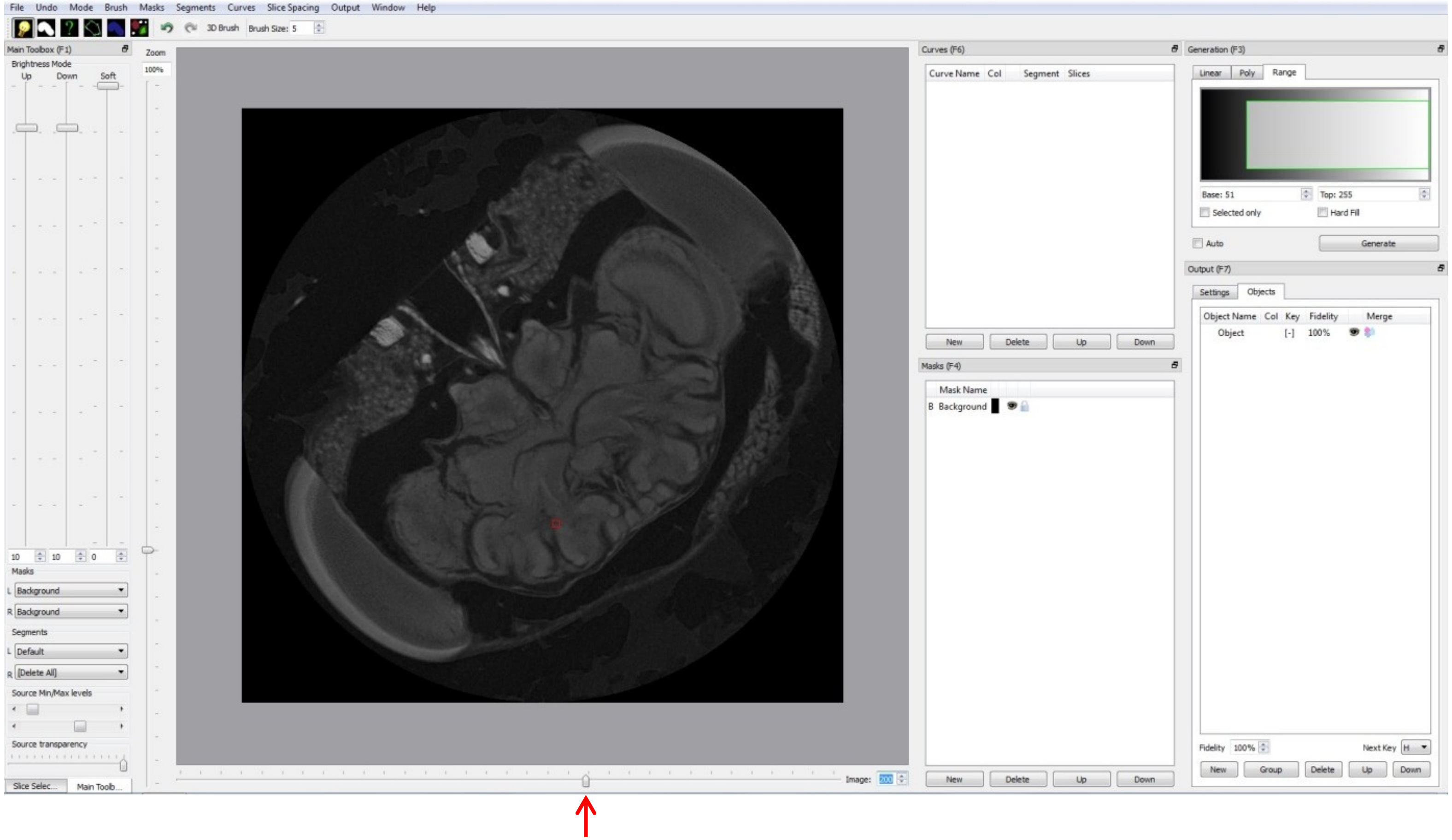

Step 2

View images in *threshold* form (space bar) which creates a black and white visualization of the image in which white pixels are “on” (treated as part of the object) and black pixels are “off” (not considered part of the object of interest and therefore excluded from further manipulation). The initial threshold view will appear all black as all pixels will at this point be set “off”.

Adjust the threshold using the *Range* tool in the *Generation* panel for a single slice from a scan in which the calyces are visible. The *Generation* panel (inside the red box) in range mode consists of a shaded strip (graded from black to white) representing the range of shades to be assigned where 0 = black and 255 = white. Using the *Base* ↑ and *Top* ↑ ‘spinboxes’ the *Base* and *Top* values representing the range on the scale covered (0 – 255) can be altered. Adjust the *Base* level in the *Range* mode of generation to achieve the best ratio of white “on” pixels comprising the calyces to black “off” pixels for the surrounding tissues. Thresholding can be achieved by manually judging the optimum levels by eye (this method is suggested for single sample analysis) or by assigning levels based on histograms of pixel intensities (suggested for multiple samples for comparative analysis). First outline the criteria your threshold should fulfil, for example: i) it should separate all ‘background’ material from the bee head tissue, ii) it should retain the identifying features of the calyces, (iii) and it should separate the calyces from the tissue directly surrounding them. To threshold manually; overlay the greyscale and binary threshold views (using the source transparency slide bar) to effectively trace the structures, adjusting the base level until the binary image fulfils the established thresholding criteria. Alternatively for multiple samples, using a histogram of pixel intensities establish a common point that best fulfils the threshold criteria - such as in this case; the value of the second peak - and use this to assign the *base* level to each sample.

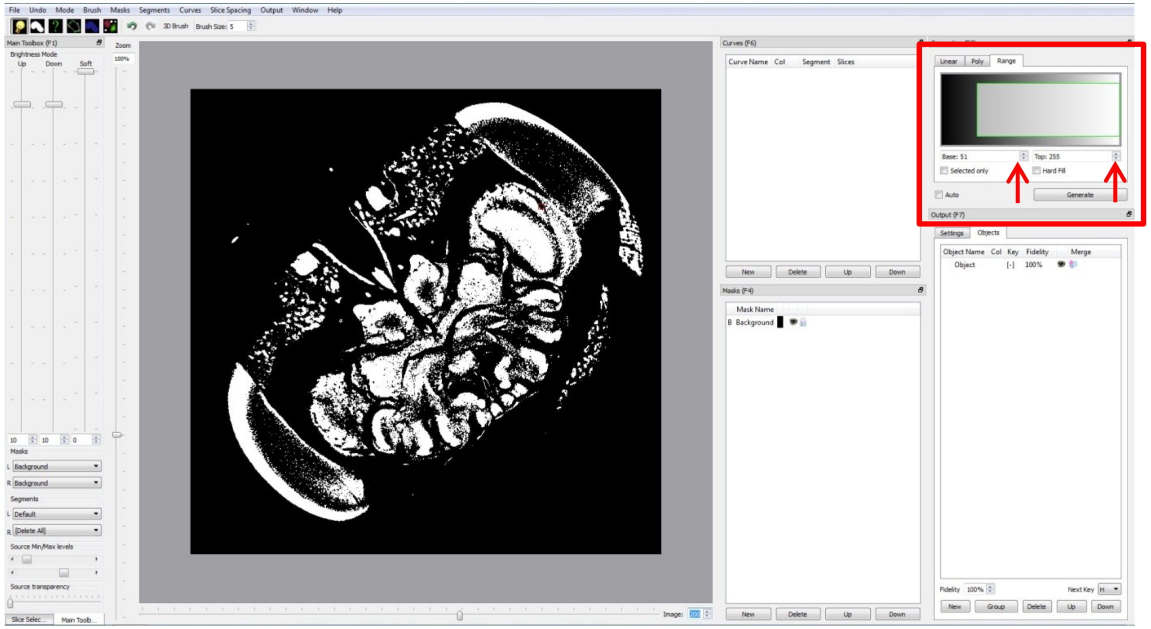

Step 3

Using either the manual or histogram based method, determine the optimum threshold (determined by the *Base* level) for 15 independent slices at 10 slice intervals across one scan. This interval spacing is selected because it corresponds well with the average number of slices per brain in which the calyces are visible. Take the average of the *Base* levels set for each of the 15 slices to obtain the base level, and hence an appropriate threshold, to apply to each individual full scan without having to go through hundreds of scan slices. The optimum threshold can then be applied to all slices of the scan using the 'generate' tool in the *Generation* panel.

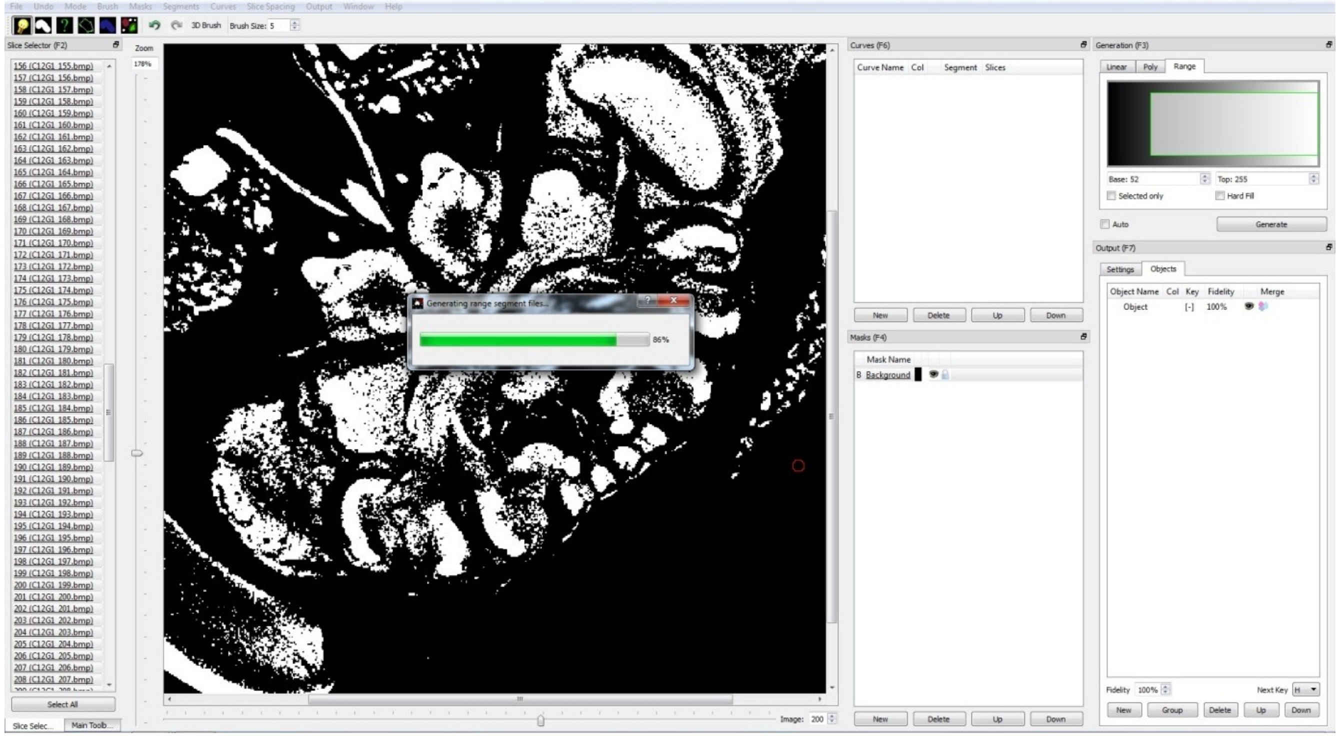

The tissues of the calyces and surrounding structures present similar grey values. This can result in a degree of superfluous tissue immediately surrounding the calyces also being included into the “on” voxels with the optimum threshold, and so into the object of interest. However such superfluous tissue can be excluded at a later stage.

Step 4

Working in the threshold view, select a central slice of the scan as a starting point, as the tissue of interest the calyces can be identified most prominently in the slices in this region of the scan, and then select ‘Curves’ Mode .

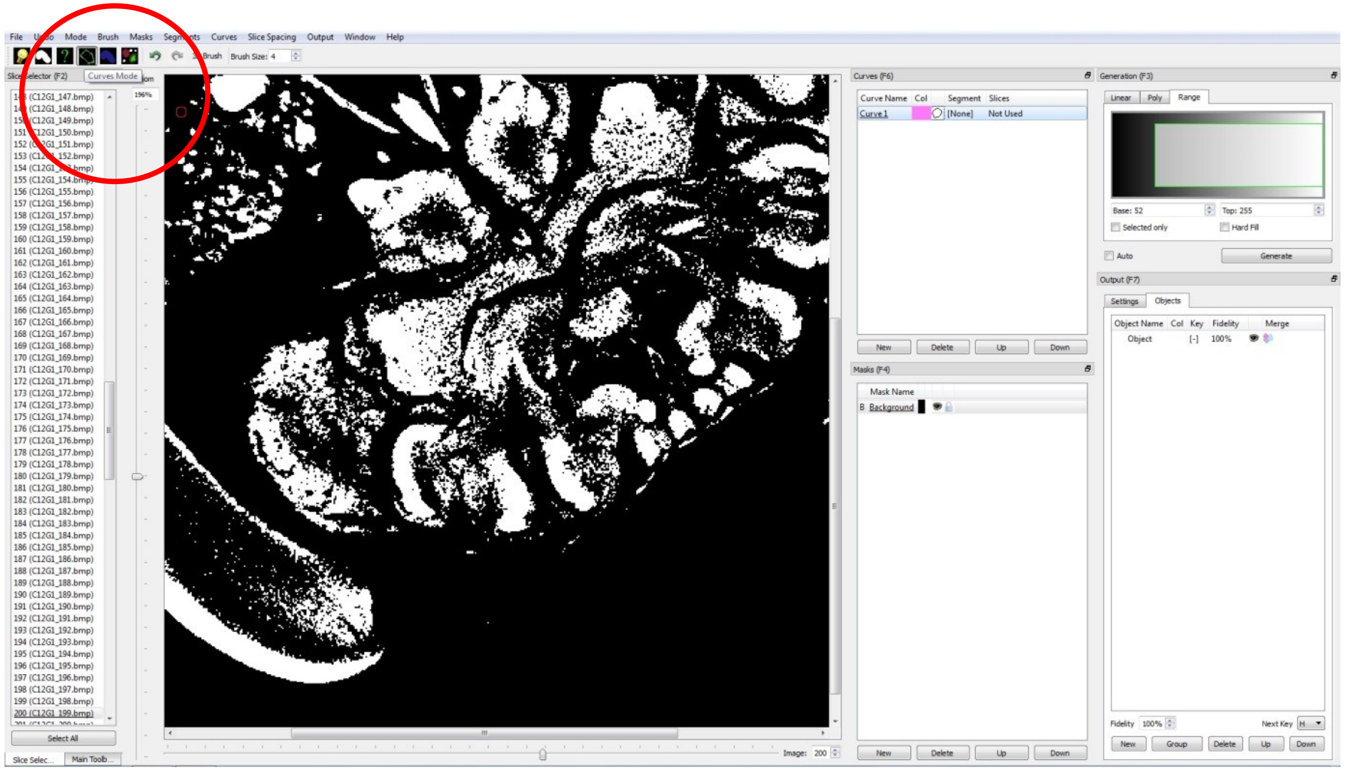

In the *curves* panel create a new curve by selecting “New” in curves panel. Then set to the closed curve option by clicking the symbol to the closed loop option indicated by the black arrow.

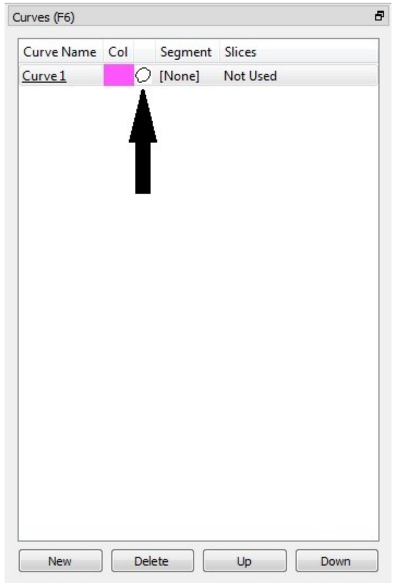

Step 5

Describe a looped spline around the calyces using the closed curve option. A *curve* can be placed around two calyces at a time (the left medial and the lateral calyces together or, right medial and lateral calyces together). To form Curve 1, a new curve is placed by pressing the "=" key (initial curve placement ◯). Each new curve initially has four *nodes* (moveable points along the curve that determine its shape).

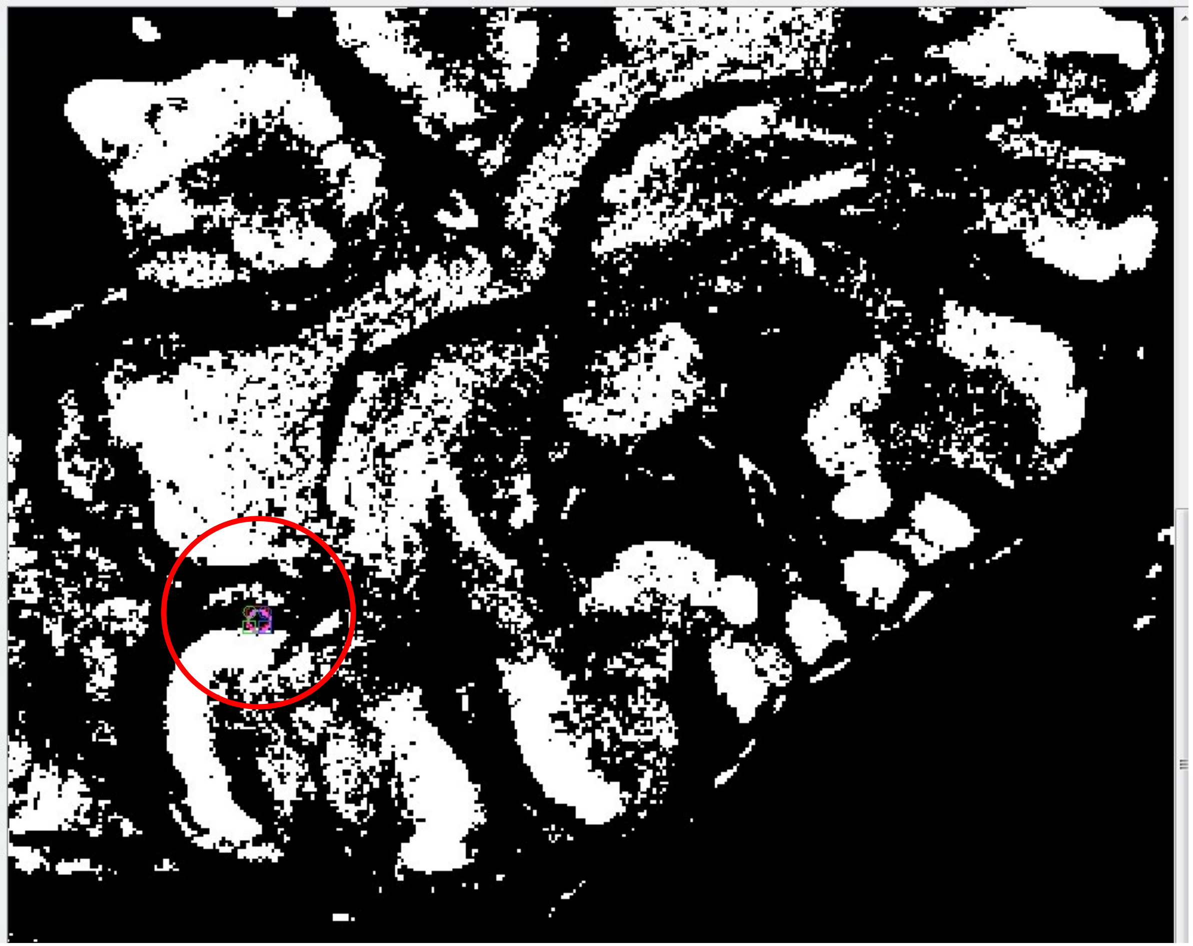

Step 6

These four nodes can then be individually moved to encircle the calyces by using your mouse to drag the cursor to place them in the appropriate positions as shown by the purple loop below.

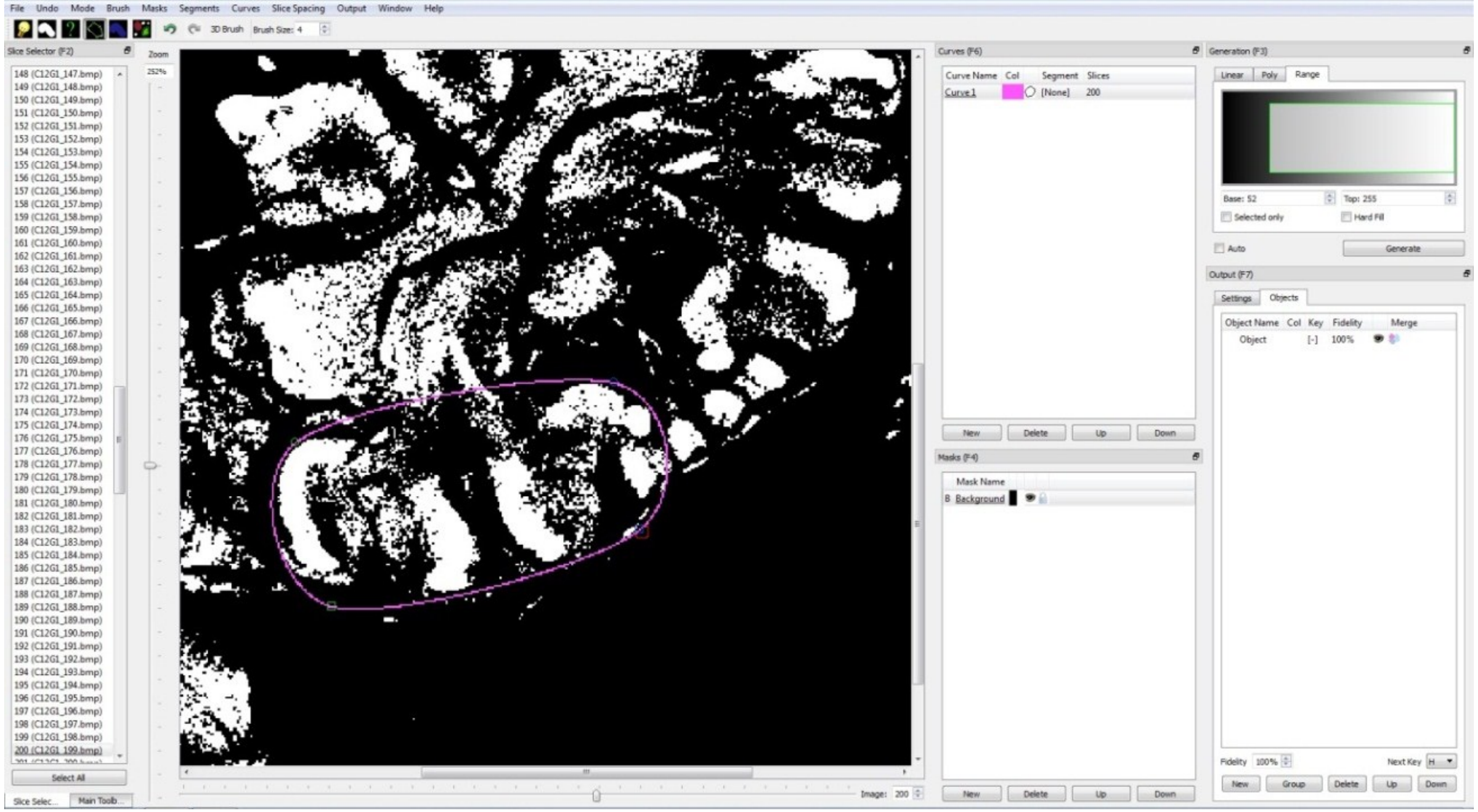

Next place a series of additional *nodes* around the calyces using the position of the cursor to determine node placement (a new node is placed by pressing the "=" key). Once the series of nodes are placed around the two calyces to form a loop, then each node can be individually moved using the cursor to closely define the shape of the calyces on that particular slice – see purple loop below.

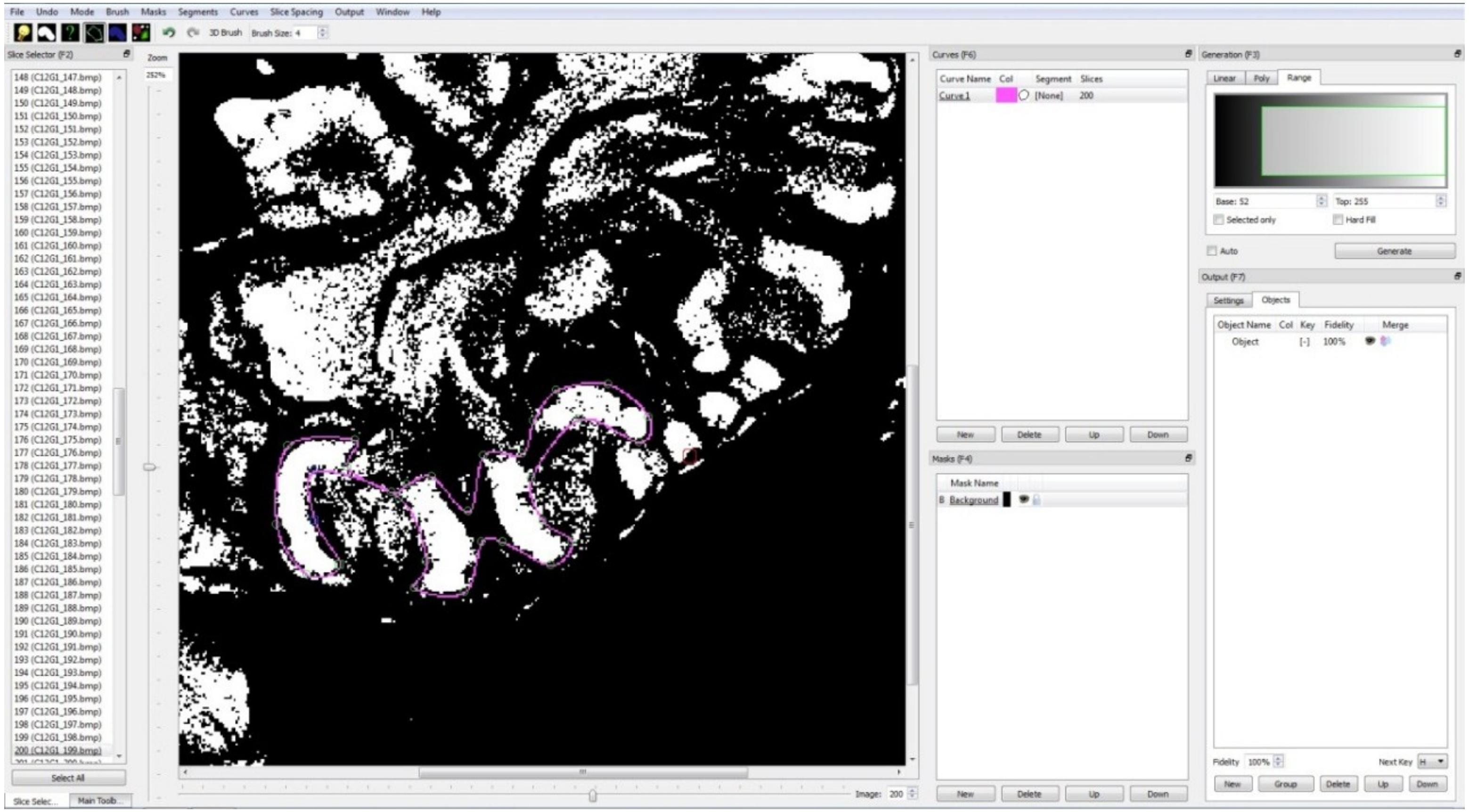

Step 7

The next slice should then be chosen - either five slices up or down from the starting slice - by scrolling along using the image bar beneath the working image. Then copy the *curve* from the first to the newly chosen slice by selecting the original slice in the *slice selector* panel ◯ and using the curves drop down menu to copy with the "copy from selected slice to current" ↑ option (or the shortcut command Ctrl + Shift + F8).

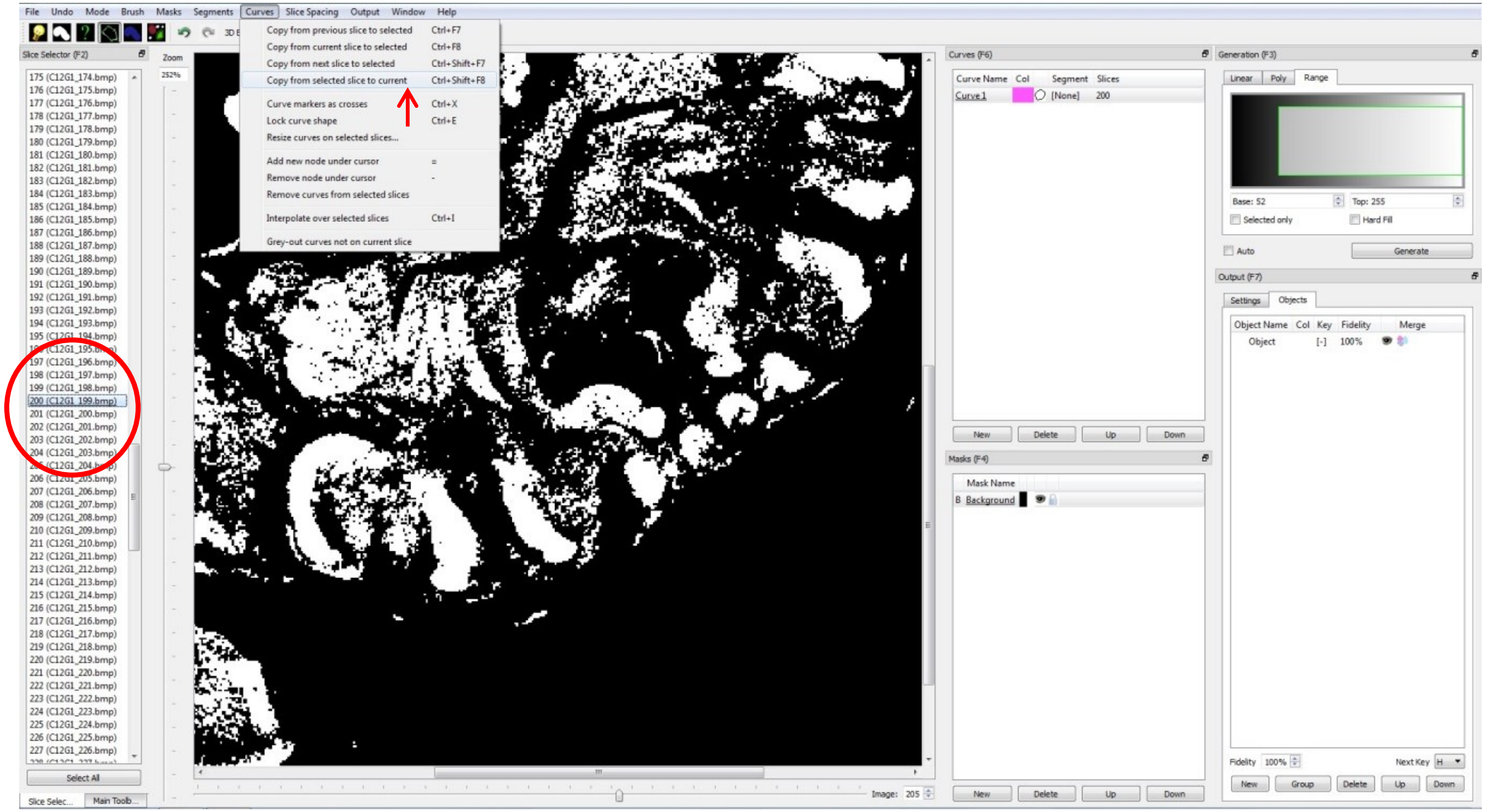

Step 8

The curve can then be adjusted to fit the shape of the calyces by manually fine tuning the position of each node in the new slice (see purple outline below).

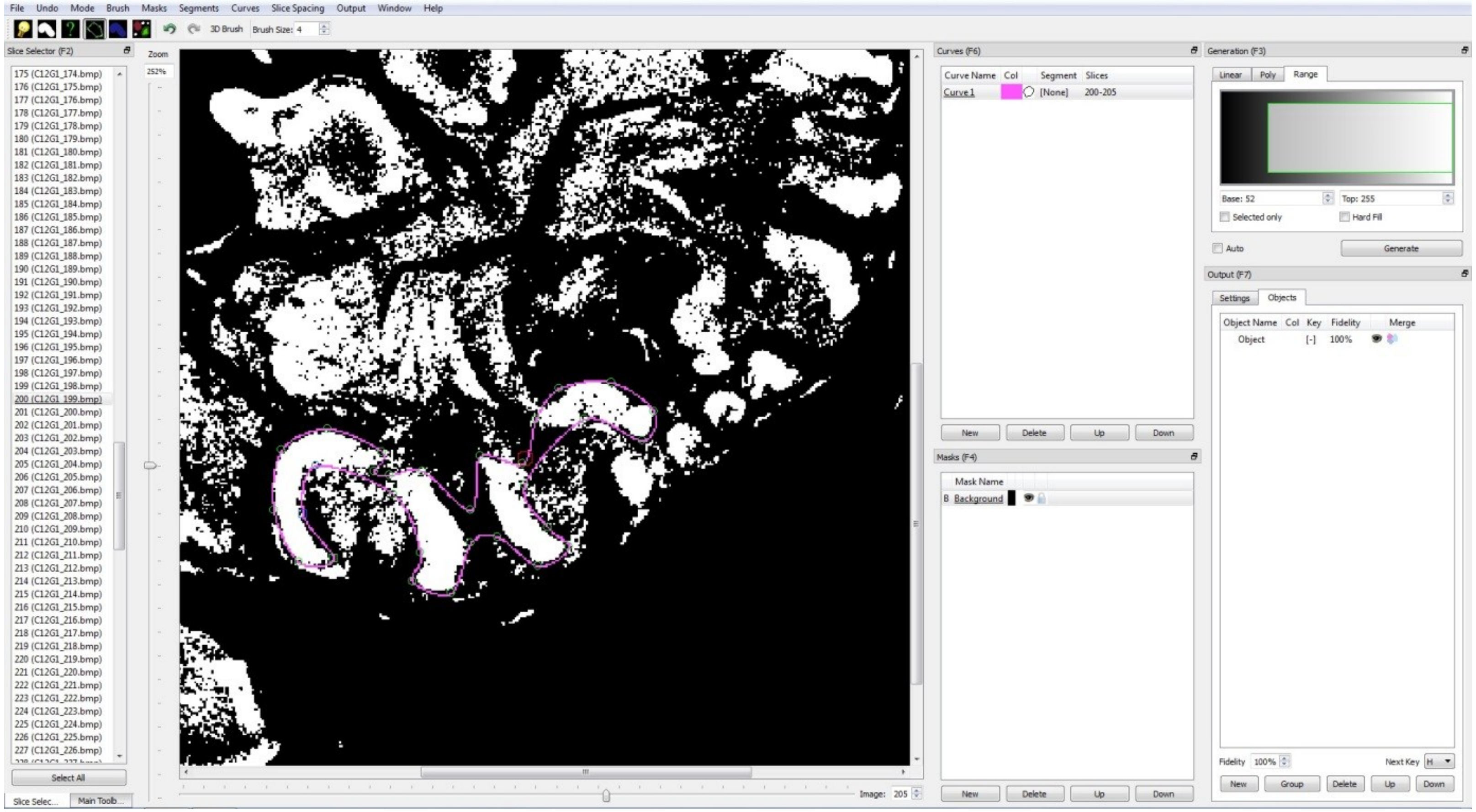

This slice is then used to copy the curve for the next slice (five slices up or down) again, readjusting the curve on the new slice to best fit the shape of the calyces.

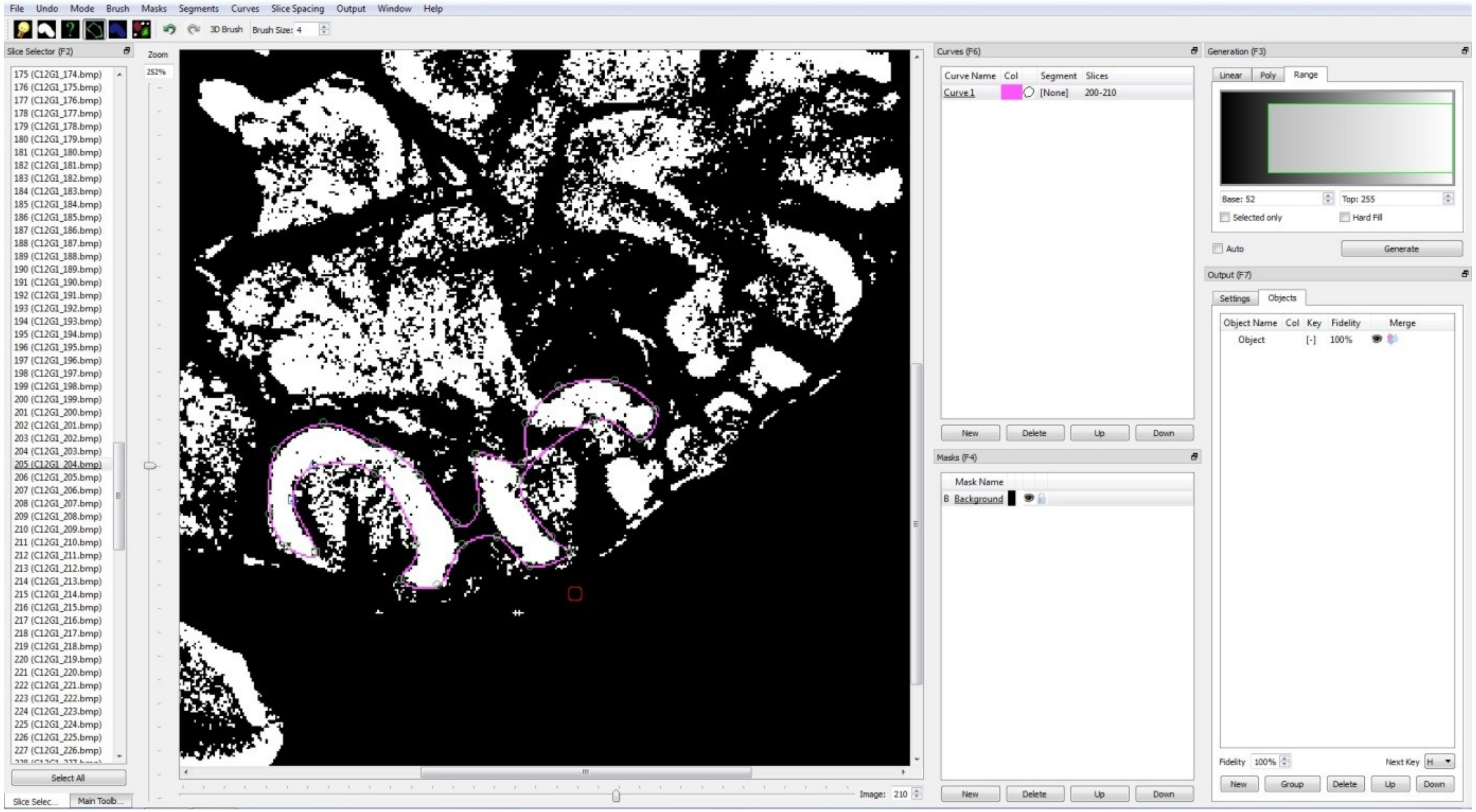

This is repeated at five slice intervals to cover all slices in which the calyces were visible, periodically checking the curve placement against the greyscale view of the calyces (pressing the space bar to change between the greyscale and threshold view) with the node positions (green spots – see below) shown over the greyscale image.

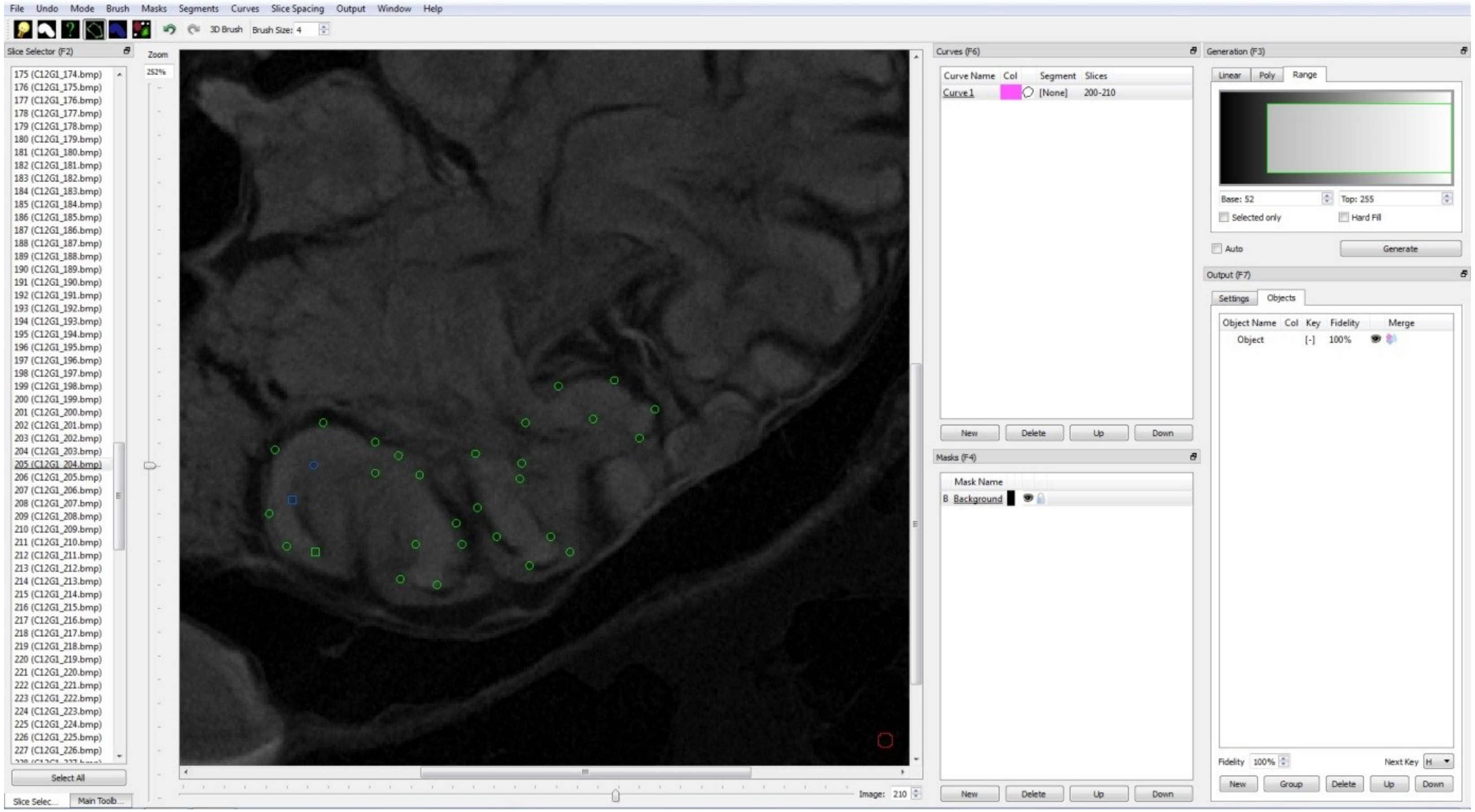

Step 9

Using the *Interpolate over selected slices* tool ↑ (in the curves drop-down menu) curves can then be placed on all slices between those selected at five slice intervals. This is done by selecting from the first to fifth for each five slice interval in the *slice selector* panel ◯.

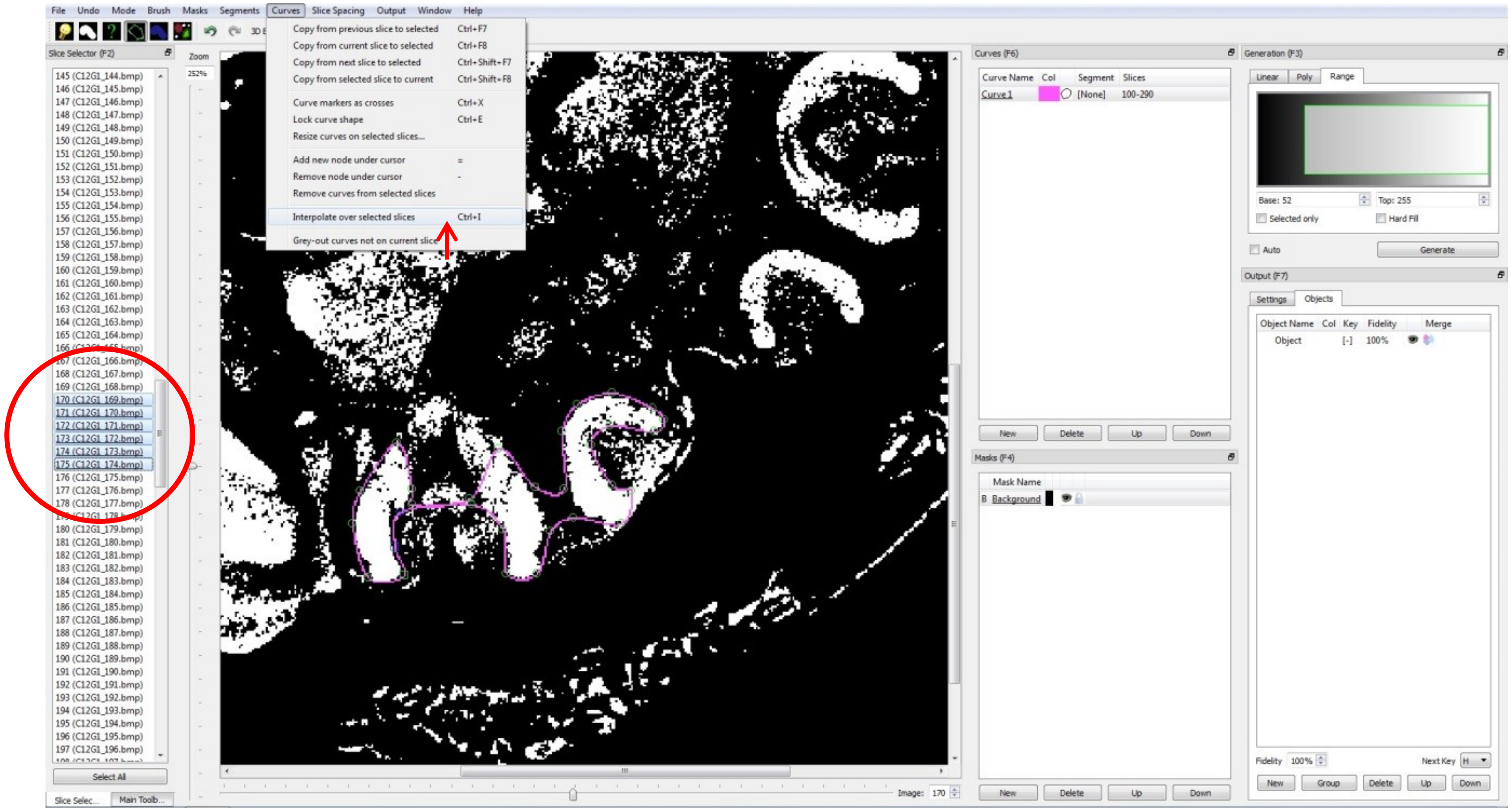

Then interpolate across selected slices (Ctrl + I) to produce gradually changing curves over all slices (see pop up highlighted by red box)

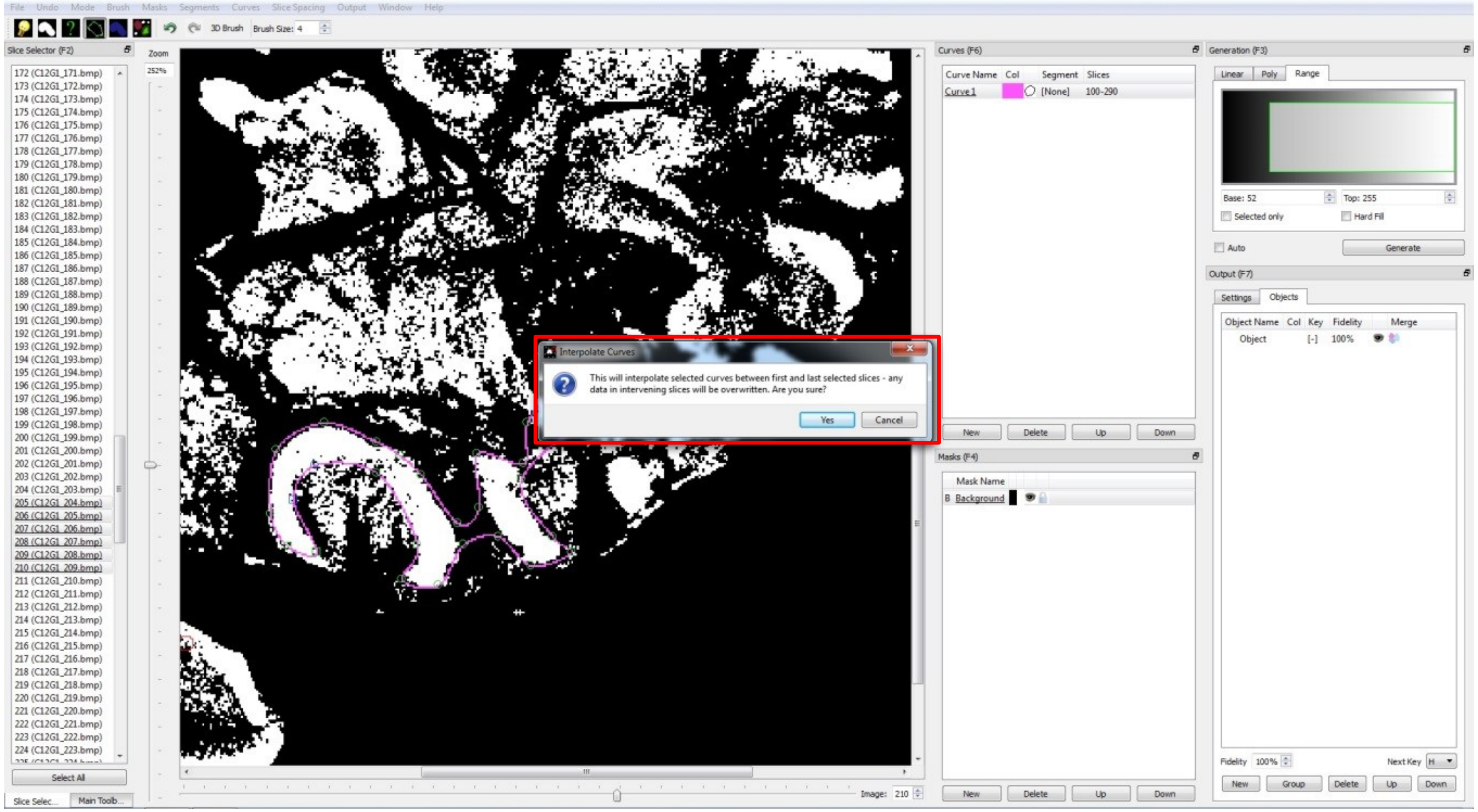

Curve 2 can then be produced for the second set of calyces (curves 1 and 2 around right calyces and left calyces respectively).

Step 10

Masks are created from the curves. Set curve one to *fill* (rather than loop) mode ↑.

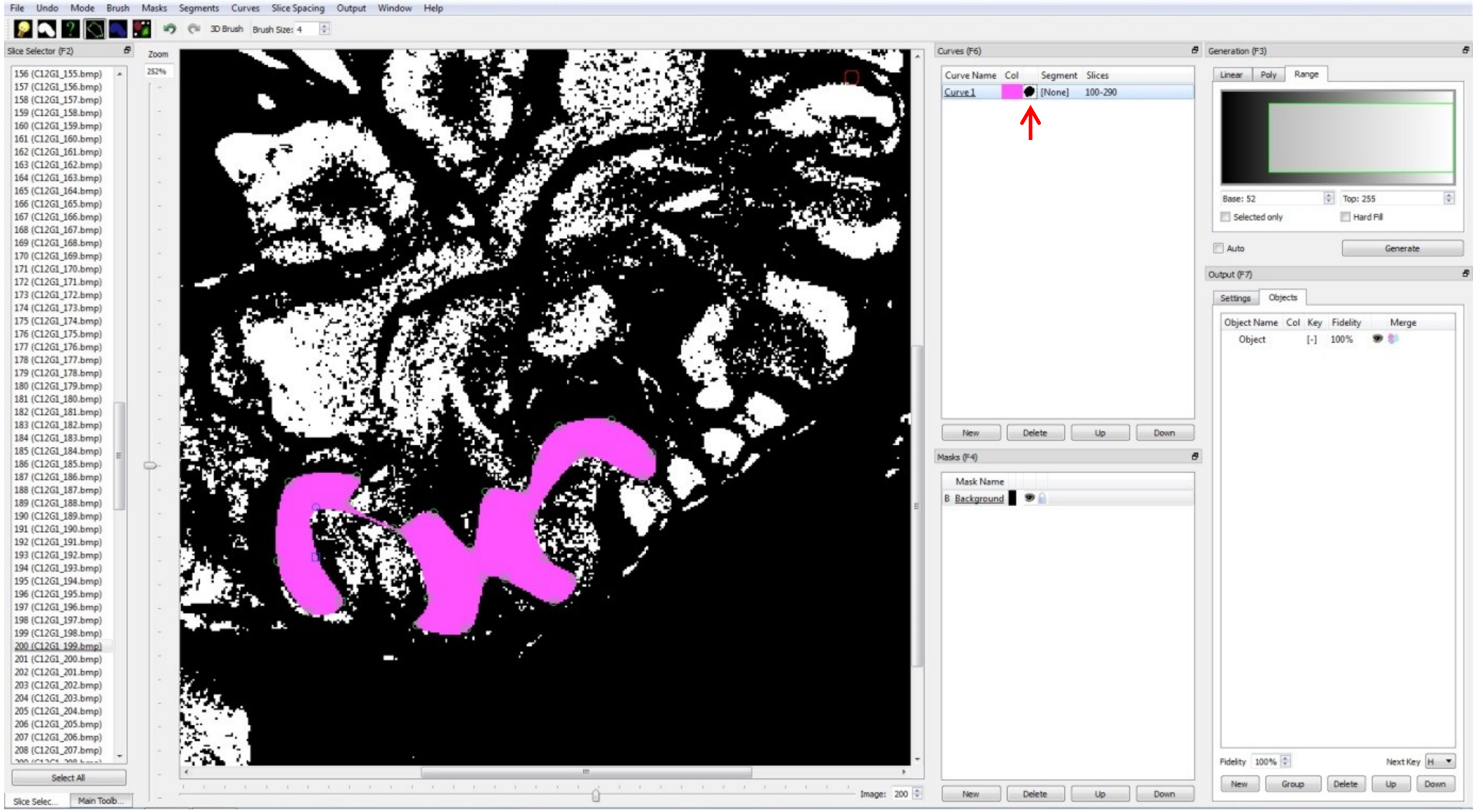

Then add a new mask in the masks panel ↑ and clicking the New option ◯ in the masks panel.

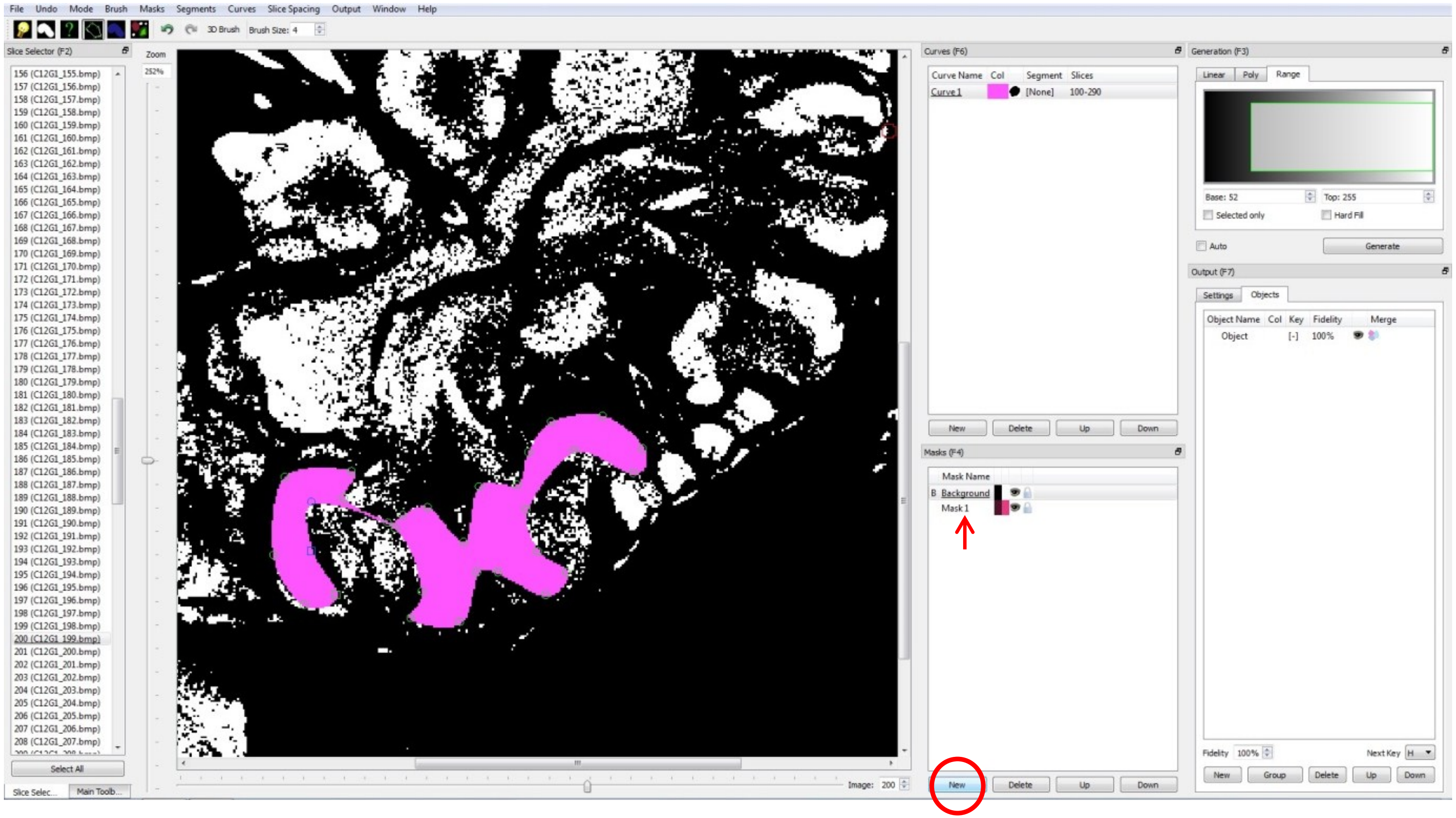

Step 11

Selecting curve 1 in the curves panel and selecting all slices in the *slice selector* panel (highlighted by red box), initiate the mask from curve function using the *mask from curve* tool ↑ (in the Masks drop-down menu) - this adds the areas covered by the curves on all slices to the new mask (mask 1). All voxels within the area designated by the curve are added to the mask.

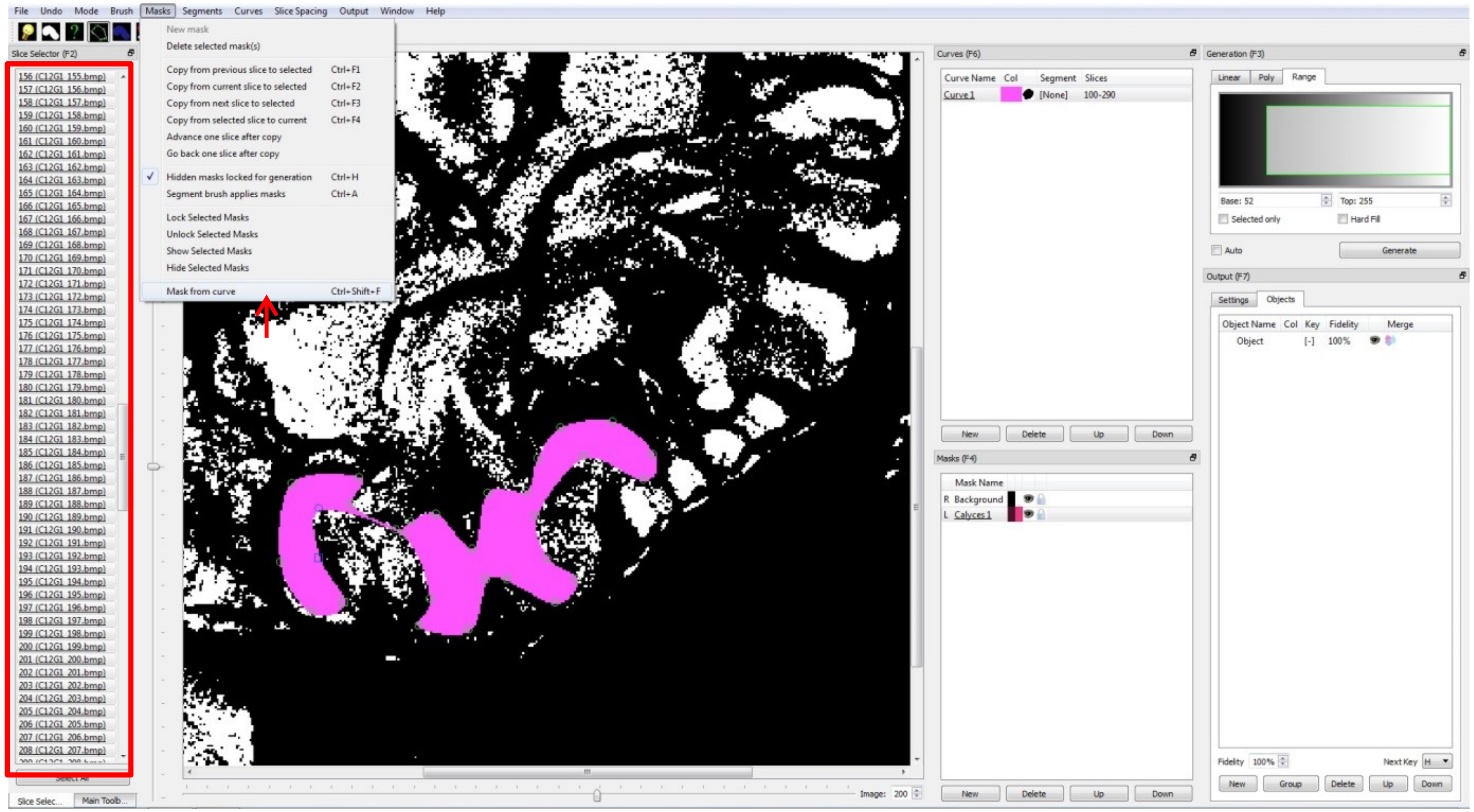

Repeat this process for curve 2 adding it to a new mask (mask 2).

Step 12

Switch to mask mode to view the mask(s) created (Ctrl-M or mask mode toggle button ↑). Each new mask can then be used to create a new *object* in the output panel by selecting the mask in the *masks* panel, then selecting “new” in the object panel. This new object comprises all the voxels within the mask(s) selected. The new object can be re-named (calyces 1).

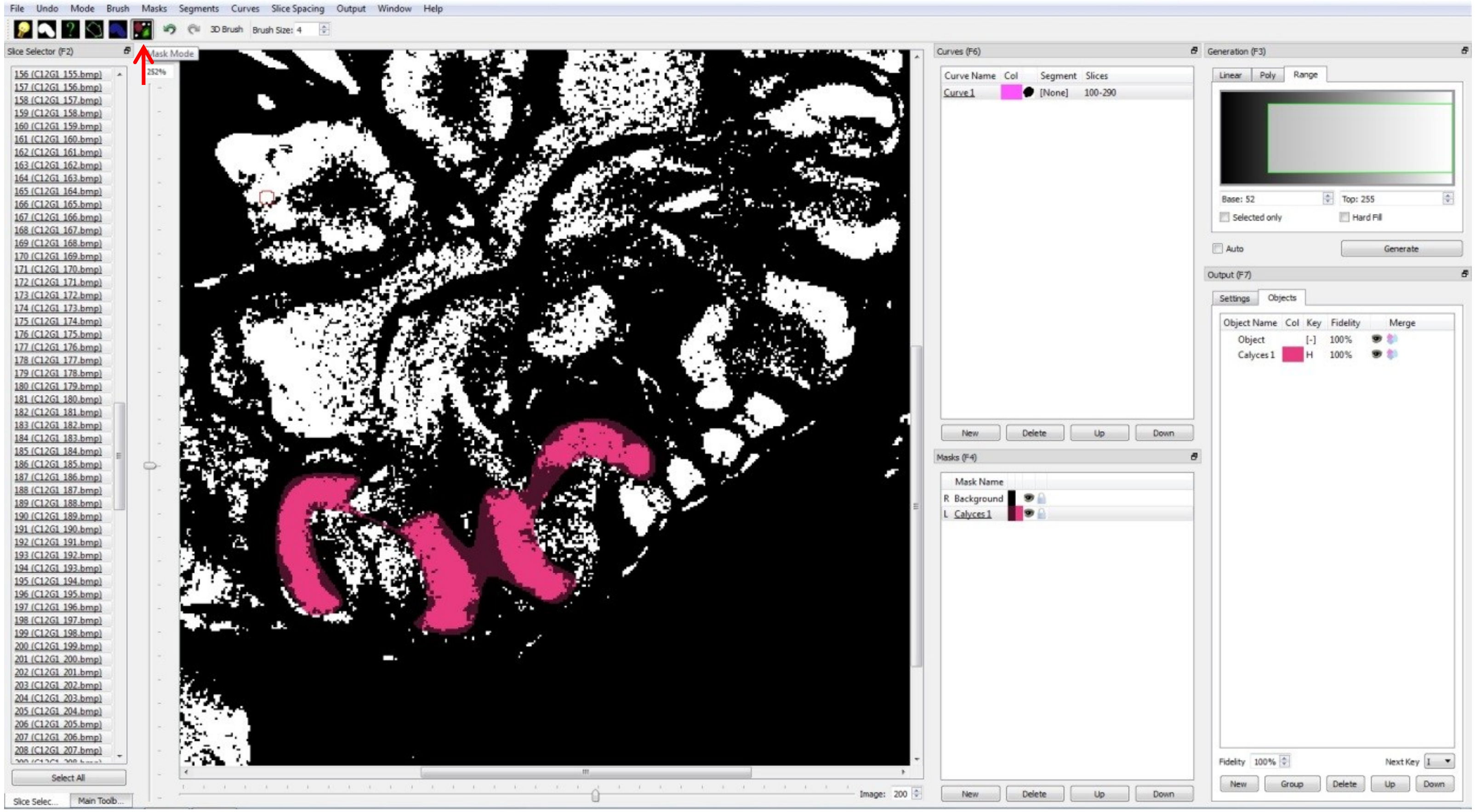

Step 13

The new object is then exported to SPIERS view by “hiding” the other objects in the objects panel (i.e. clicking the eye icon next to the object ↓, then selecting “view in SPIERS view” ↑ from the drop-down output menu).

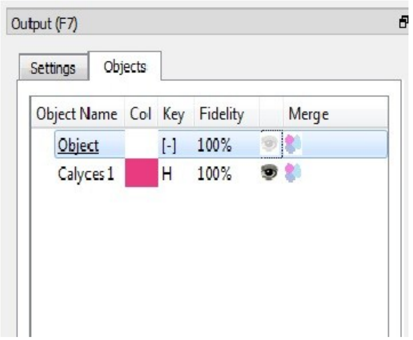

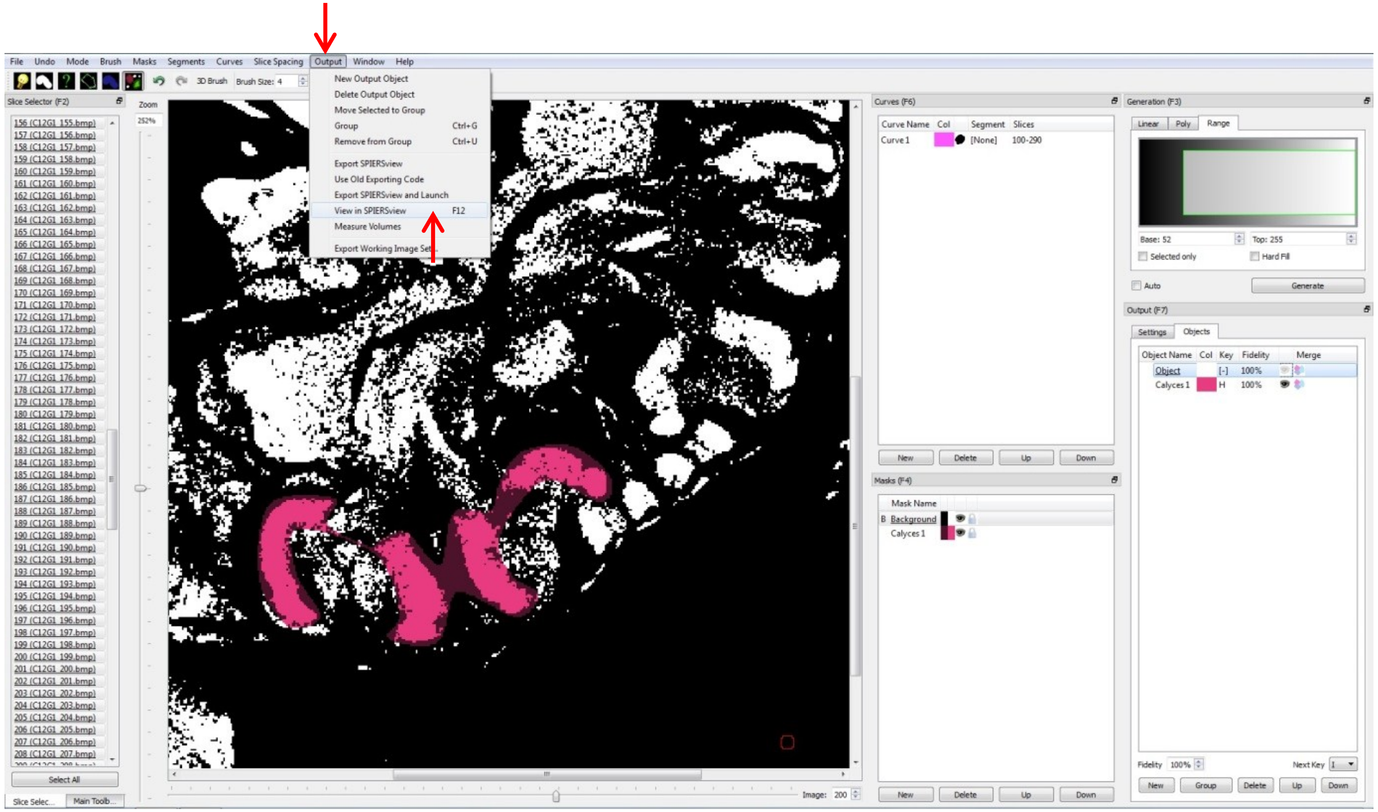

3D Reconstruction – SPIERS View

Step 14

SPIERS view reconstructs the region of the images defined by the object allowing three-dimensional viewing of the calyces independently of the rest of the brain. Using SPIERS view the calyx morphology can be visualised. In SPIERS view, apply automated processing features, “smoothing” and “island removal”, to 3-D reconstructions by selecting the object(s) calyces 1 and calyces 2 in the objects panel ◯. Then select the smoothing and Island removal options from the objects menu ↑ to set levels of each. *NB*: SPIERS view sets the colour based on their settings when exported from SPIERS edit; whilst the colours are assigned randomly you can simply change this manually by using the “colour” box in the object/mask/curve panels.

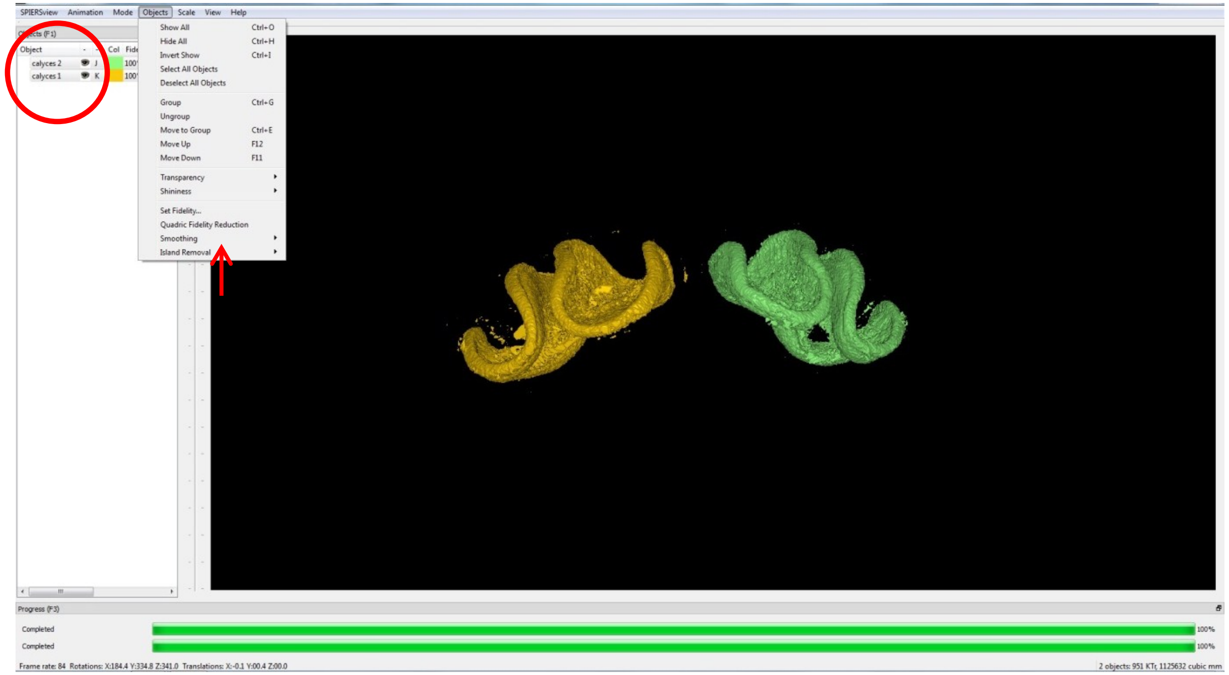

Then apply the antialiasing option (x16) to implement the smoothing and island removal levels.

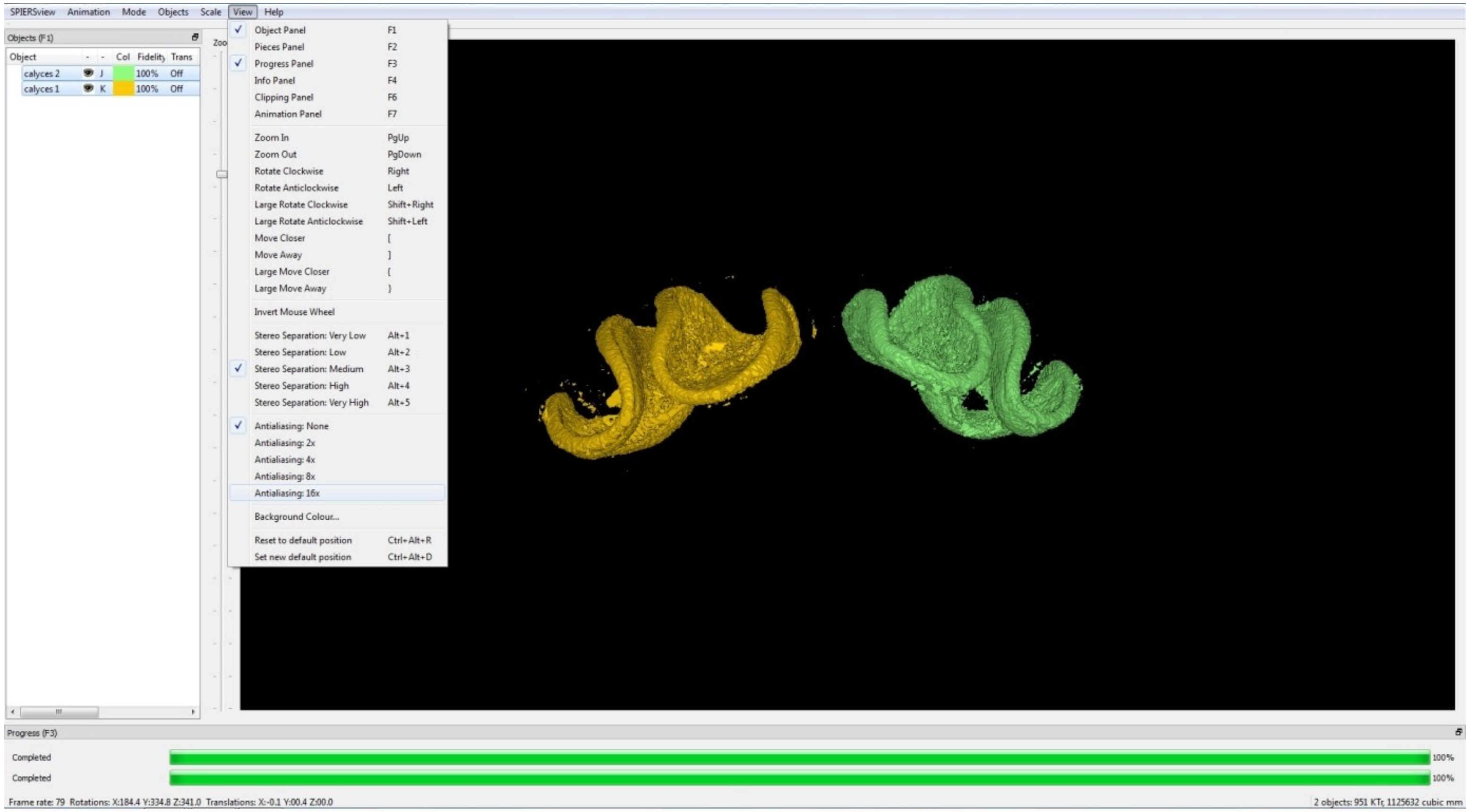

These features reduce the amount of extraneous tissue and smooth any rough edges of the objects.

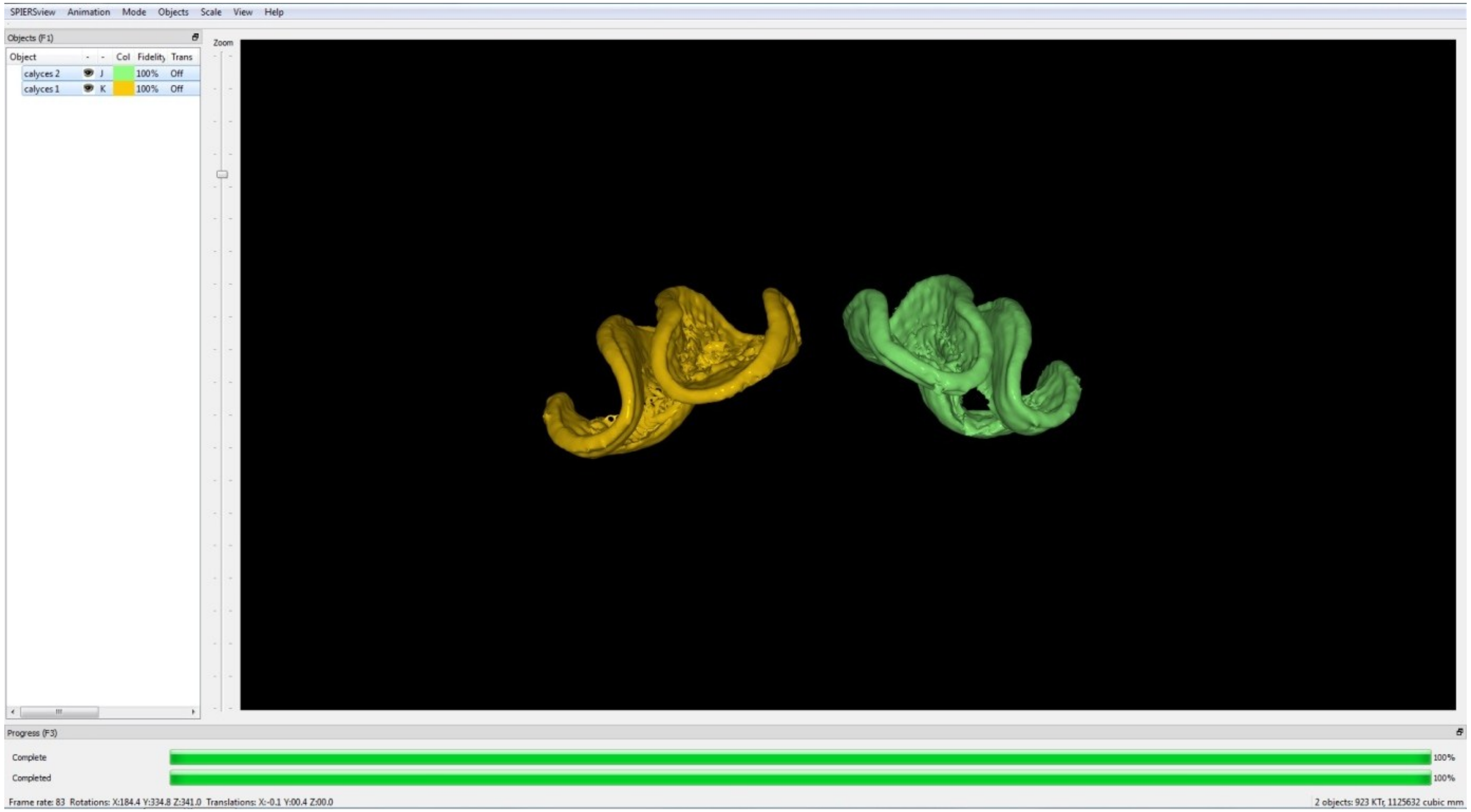
SPIERSview Animation Mode Objects Scale View Help
Objects (F1)
Object
Col
Fidelity
Trans
calyces 2
100%
Off
calyces 1
100%
Off
Zoom
Progress (F3)
Complete
100%
Completed
100%
Frame rate: 83 Rotations: X:184.4 Y:334.8 Z:341.0 Translations: X:-0.1 Y:00.4 Z:00.0
2 objects: 923 KTr, 1125632 cubic mm

Volumetric Analysis – SPIERS Edit

Step 15

Volumetric analysis of each independent object can be carried out in SPIERS edit. The *measure volumes* ↑ tool in the Output drop-down menu is selected, with all objects in the objects panel “unhidden”. This tool calculates the volume of each individual object in the Object panel (e.g. calyces) and the total volume of the sample (e.g. the scanned head sample).

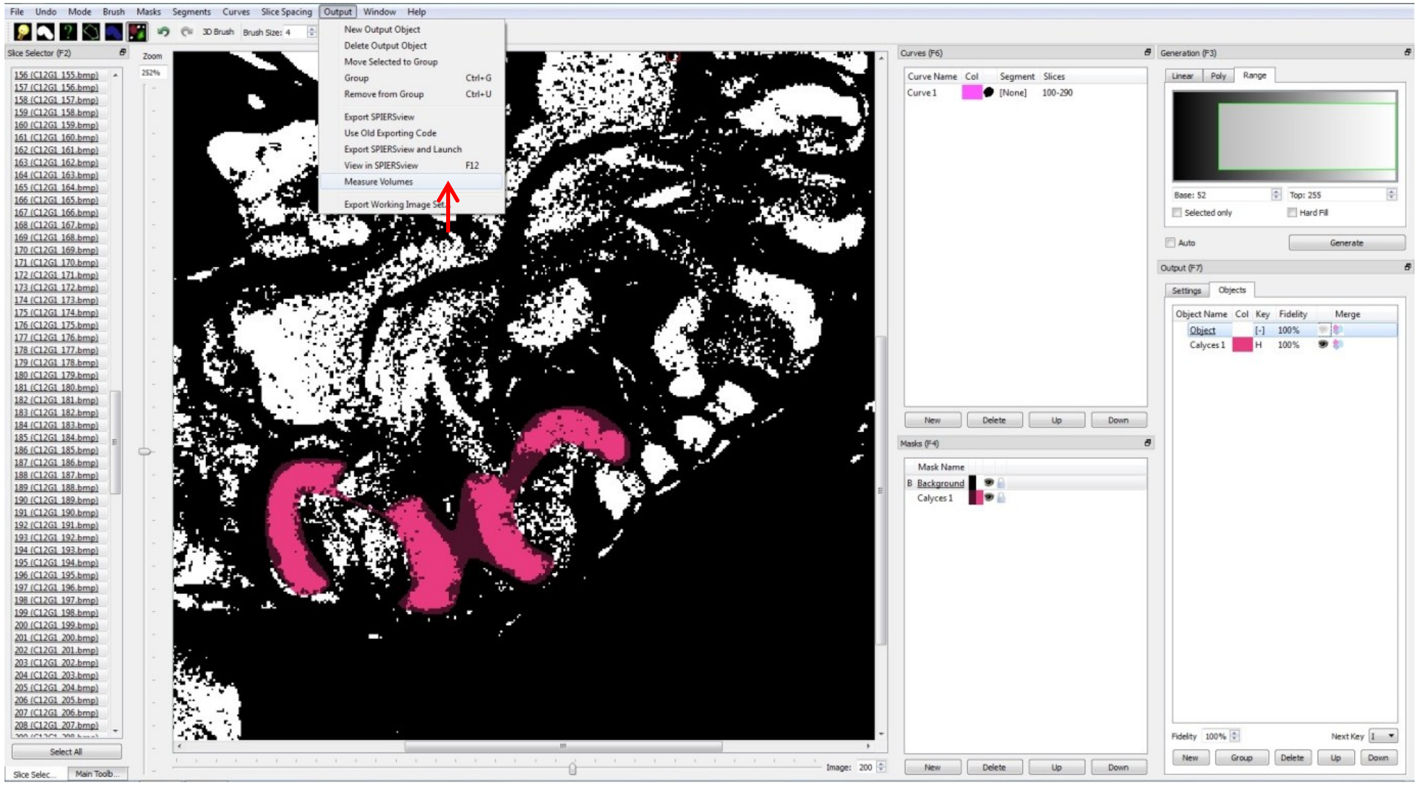

The volumes of objects are given both as the number of voxels and also as a percentage of the total tissue volume in the sample ◯.

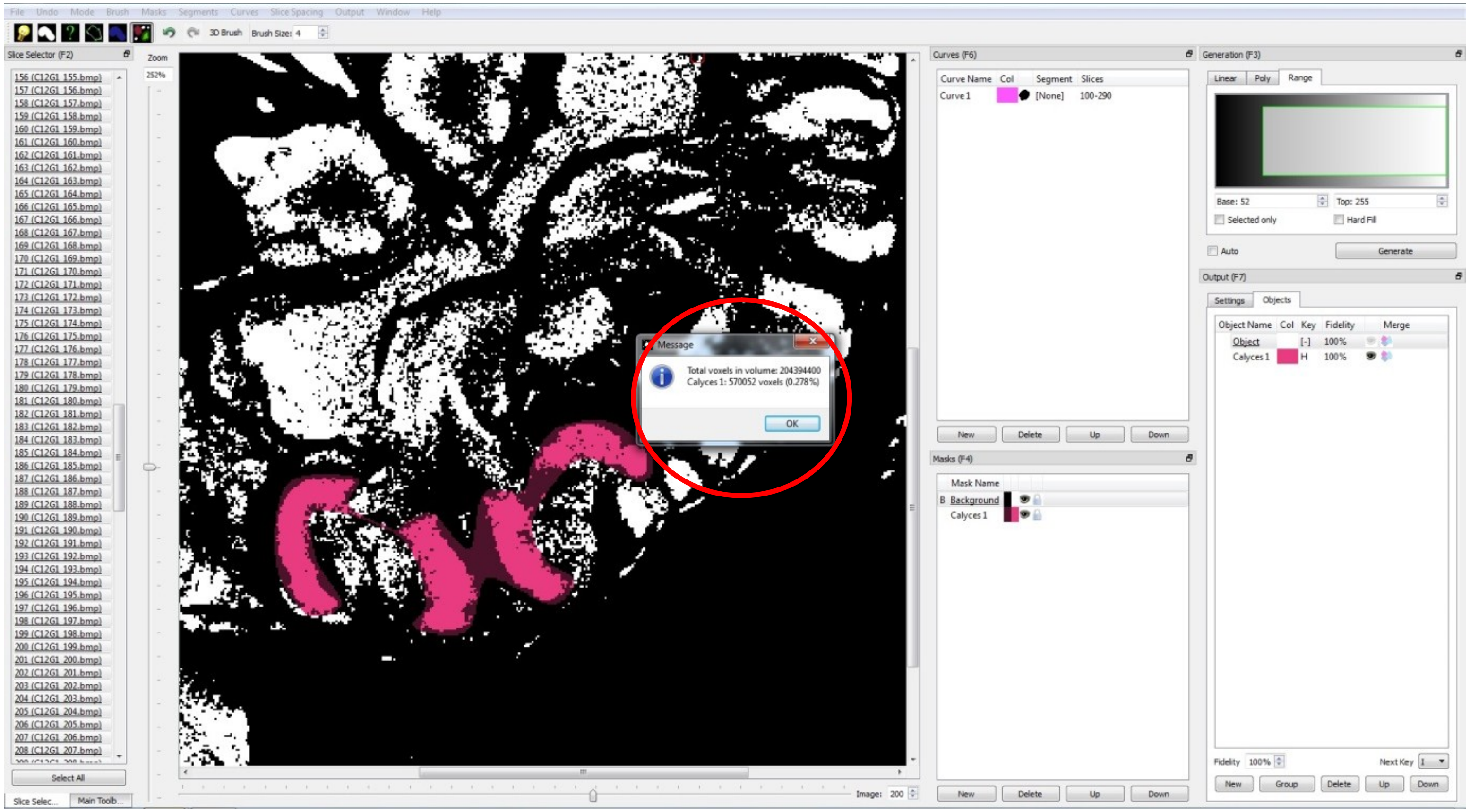
File Undo Mode Brush Masks Segments Curves Slice Spacing Output Window Help
3D Brush
Brush Size: 4
Slice Selector (F2)
Zoom
Select All
Curves (F6)
Curve Name Col Segment Slices
Curve 1 [None] 100-290
New Delete Up Down
Masks (F4)
Mask Name
Background
Calyces 1
Generation (F3)
Linear Poly Range
Base: 52
Top: 255
Selected only
Hard Fill
Auto
Generate
Output (F7)
Settings Objects
Object Name Col Key Fidelity Merge
Object [-] 100%
Calyces 1 H 100%
Fidelity 100%
Next Key
New Group Delete Up Down
Image: 200
Message
Total voxels in volume: 204394400
Calyces 1: 570052 voxels (0.278%)
OK
